# Collecting wild *Miscanthus* germplasm in Asia for crop improvement and conservation in Europe whilst adhering to the guidelines of the United Nations’ Convention on Biological Diversity

**DOI:** 10.1093/aob/mcy231

**Published:** 2018-12-22

**Authors:** Lin S Huang, Richard Flavell, Iain S Donnison, Yu-Chung Chiang, Astley Hastings, Charlotte Hayes, Chris Heidt, Hao Hong, Tsai-Wen Hsu, Mervyn Humphreys, Julian Jackson, John Norris, Kai-Uwe Schwarz, Michael Squance, Timothy Swaller, Ian David Thomas, Wilfriede Van Assche, Qingguo Xi, Toshihiko Yamada, Sue Youell, John Clifton-Brown

**Affiliations:** 1 Institute of Biological, Environmental and Rural Sciences (IBERS), Aberystwyth University, Plas Gogerddan, Aberystwyth, UK; 2 CERES Inc., 1535 Rancho Conejo Blvd, Thousand Oaks, CA, USA; 3 Department of Biological Sciences, National Sun-Yat-Sen University, Kaohsiung, Taiwan; 4 Institute of Biological and Environmental Science, University of Aberdeen, Aberdeen, UK; 5 The Guangdong Association of Grass and Environment, Tianhe District, Guangzhou, China; 6 Taiwan Endemic Species Research Institute (TESRI), Chi-Chi, Nantou, Taiwan; 7 Department for Environment, Food and Rural Affairs, Nobel House, 17 Smith Square, London, UK; 8 Julius Kuhn Institut (JKI) Bundesforschungsinstitut für Kulturpflanzen, Institute for Crop and Soil Science, Bundesallee, Braunschweig, Germany; 9 Dongying Agricultural Institute, Jiaozhoulu 383, Dongying, Shandong Province, China; 10 Field Science Centre for the Northern Biosphere, Hokkaido University, Sapporo, Japan

**Keywords:** *Miscanthus*, bioenergy, plant breeding, germplasm collection, germplasm evaluation, Convention on Biological Diversity, CBD, the Nagoya Protocol, conservation, biodiversity, Bonn Guidelines, ethical use of genetic resources

## Abstract

**Background and Aims:**

Germplasm with diverse, agronomically relevant traits forms the foundation of a successful plant breeding programme. Since 1993, the United Nations has been advocating the implementation of the Convention on Biological Diversity (CBD) and the subsequent 2002 Bonn Guidelines as international best practice on germplasm collection and use. In 2006, a European team made an expedition to Asia to collect wild germplasm of *Miscanthus*, a C_4_ perennial rhizomatous grass, for breeding an environmentally adaptable, resilient and high-yielding bioenergy crop. We outline general aspects of germplasm collection, conservation, breeding and biomass production evaluation while following the CBD’s guidelines, respecting biodiversity and conservation needs, and the ethical use of genetic resources.

**Methods:**

Effective protocols, quarantine, methods for collecting seed and rhizomes, and a genebank for conservation were established. Versatile informatics and database architecture were used to assist in selection, flowering synchronization, crossing, evaluation, phenotyping and data integration. Approaches were formulated to comply with the CBD guidelines.

**Key Results:**

A total of 303 accessions of *M. sinensis*, *M. sacchariflorus* and *M. floridulus* were collected from 158 geographically and environmentally diverse locations. These species were shown to accumulate different amounts of aerial biomass due to combinations of stem count, height and thickness. Progeny from one interspecies cross accumulated more biomass in early trials and has shown double the yield performance in years 3–4 compared with the existing commercial cultivar *M. × giganteus*. An example of an F_1_ hybrid has already demonstrated the long-term potential of exploiting this collection for a breeding programme.

**Conclusions:**

By conforming to the CBD principles, the authors’ international collaboration provides a practical example of implementing the CBD. The collection widened the genetic diversity of *Miscanthus* available to allow for breeding of novel hybrids that exhibit more diverse traits to increase yield and resilience for growth on marginal land and in climate-challenged environments.

## INTRODUCTION

Climate change mitigation requires replacement of fossil fuel-derived energy and products with renewable equivalents. Biomass from bioenergy crops, because it can be used for liquid transport fuels and can be combined with carbon capture and storage to create negative CO_2_ emissions, is considered to be a key part of the renewable energy mix. To deliver maximum benefits, bioenergy crops need to be high yielding with a low requirement for inputs of nutrients and water, to be able to grow in a wide range of soils and climates including on marginal land, and to have minimal impact on food production (The Royal Society, 2009; Flavell *et al.*, 2011; Valentine *et al.*, 2012).

The *Miscanthus* genus contains a number of highly productive perennial grasses (Jones and Walsh, 2001) which use the C_4_ photosynthetic pathway. Wild *Miscanthus* germplasm is adapted to a very wide geographical area from the tropics in South-east Asia through to Siberia. *Miscanthus* species can potentially generate high biomass yields over successive years with minimal inputs of energy-demanding nitrogen fertilizers, and many show extreme cold tolerance, efficient carbon fixation and high water use efficiency (Clifton-Brown, 2000; Clifton-Brown *et al.*, 2001*a*; Heaton *et al*., 2004, 2010; Hastings *et al*., 2009, 2014). One naturally occurring triploid genotype, *M. × giganteus* (type specimen ‘1993-1780’, Greef and Deuter, 1993; Hodkinson and Renvoize, 2001) has been cultivated as a commercial bioenergy crop in recent decades and has produced high yields on many sites in Europe (Lewandowski *et al.*, 2000; Clifton-Brown *et al.*, 2001*b*) and the USA (Dohleman and Long, 2009). It is, however, expensive to establish as it can only be clonally propagated via rhizomes and commonly suffers from patchiness in establishment. These have economic and pest and disease implications for large-scale cultivation and hence the delivery of environmental impact (Lewandowski *et al.*, 2000; Zimmermann *et al.*, 2013; Dauber *et al.*, 2015). Therefore, further development of the crop is needed in order to increase genetic diversity, improve yield, quality and resilience, enable growth in diverse environments to optimize the efficiency of land use, and improve yield and resilience to mitigate climate change and extreme weather events. However, to create improved, high-yielding and locally adapted varieties through plant breeding requires diverse and extensive germplasm.

Prior to 2006, the Institute of Biological, Environmental and Rural Sciences (IBERS) of Aberystwyth University (AU) had gathered 244 genotypes comprising 187 *M. sinensis*, 35 *M. sacchariflorus* and 22 interspecific hybrids, and including the *M.* × *giganteus* genotype as a control for a replicated trait trial (Clifton-Brown *et al.*, 2008; Jensen *et al.*, 2011*a*). They were assembled from collections made by Kew (UK), ADAS (UK), historical European collections and European-funded projects including EMI (European *Miscanthus* Improvement), FAIR 3 CT-96–1392 co-ordinated by the University of Hohenheim, Germany (Clifton-Brown *et al.*, 2001*b*) and BIOMIS co-ordinated by Plant Research International (PRI) in Wageningen, The Netherlands (Atienza *et al.*, 2002). Many of these plants were selected by taxonomists seeking botanical diversity (Hodkinson and Renvoize, 2001; Hodkinson *et al.*, 2002; Clifton-Brown *et al.*, 2011), rather than the genetic/agronomic diversity in traits needed for a crop breeding programme. The collection also included ornamental cultivars from garden centres embracing diploid, triploid and tetraploid genotypes. This collection has been studied and utilized extensively in research programmes at IBERS over the last decade, including the application of genomic and phenomic information useful for breeding (Clifton-Brown *et al.*, 2008; Hastings *et al*., 2009, 2014; Allison *et al.*, 2011; Jensen *et al.*, 2011*a*, *b*; Robson *et al*., 2011*a*, *b*, 2012, 2013*a*, *b*; Slavov *et al.*, 2013). A more recent US collection which focused on collecting *M. sinensis* for breeding was characterized and used to demonstrate the genetic diversity and population structure of *M. sinensis* collected from Japan, China and South Korea (Clark *et al.*, 2014; Sacks, 2016).

Since the entry into force of the United Nations Convention on Biological Diversity (CBD) in 1993, any new genetic resources collected need to abide by the CBD’s principles of access and benefit sharing where this is required by national legislation (United Nations, 1993). These principles were further elaborated in the voluntary Bonn guidelines on access to genetic resources and fair and equitable sharing of the benefits arising out of their utilization (United Nations, 2002), agreed in 2002, and the Nagoya protocol on access to genetic resources and the fair and equitable sharing of benefits arising from their utilization to the Convention on Biological Diversity adopted in 2010 (Nagoya Protocol, 2010). This is transforming the way germplasm is accessed and utilized, and is having a profound impact on conservation, biodiversity and the ethical use of genetic resources on a global scale.

The three objectives of the CBD are (1) to pursue ‘the conservation of biological diversity’; (2) to pursue ‘the sustainable use of the components of biological diversity’ through national and international co-operation; and (3) to ensure the fair and equitable sharing of the benefits arising out of the utilization of genetic resources. The Nagoya Protocol, which was adopted on 29 October 2010 in Nagoya, Japan, is a supplementary agreement to the CBD. It provides a transparent legal framework for the effective implementation of CBD’s third objective ‘access and benefit sharing (ABS)’. Further descriptions on the 1993 CBD, 2010 Nagoya Protocol and 2014 EU Regulation (EU) No. 511/2014 (Defra, 2014; European Union, 2014, 2015) on compliance measures for users from the Nagoya Protocol in the European Union can be found in Supplementary Data S1.

To expand the historical European collection, the UK Department for Environment, Food and Rural Affairs (Defra) commissioned IBERS at AU to lead a *Miscanthus* collection in China, Japan and Taiwan for incorporation into the UK *Miscanthus* breeding programme while adopting the principles and guidelines of the CBD. This *Miscanthus* germplasm collection (Asia-2006) aimed to increase the genetic diversity for breeding an environmentally adaptable and resilient, high-yielding bioenergy crop suitable for the UK and Europe. Another important objective was to collect diploid *M. sinensis* and tetraploid *M. sacchariflorus* that could be used as parents to breed high-yielding sterile triploid hybrids to address potential concern about the invasiveness (Quinn *et al.*, 2010) arising from growing large acreages of *Miscanthus* outside its native Asia. It is important that this germplasm collection needed to be consistent with the CBD principles, safeguarding biodiversity, respecting conservation needs and the ethical and sustainable use of the genetic resources.

Aspects of biomass production from a selection of the genotypes collected, with an example of early heterotic growth in an interspecies cross based on parents from different countries, are also included herein as an illustration of the potential gain that can be achieved from such a collection.

## MATERIALS AND METHODS

**Fig. 1.**
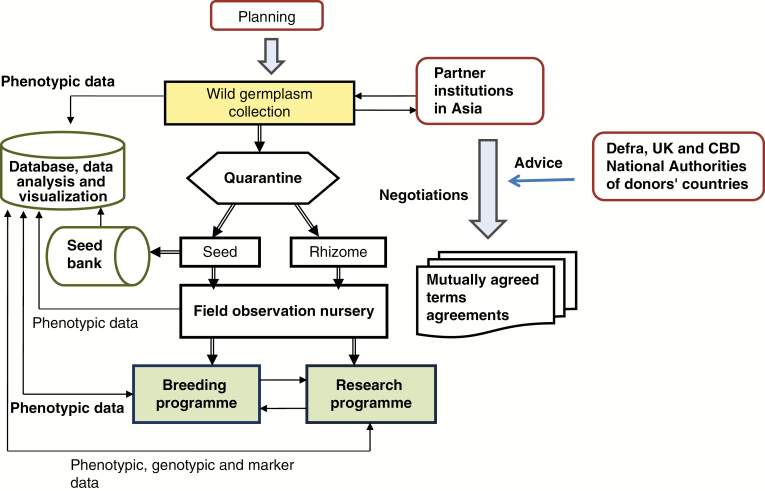
Schematic depicting the steps taken in bringing wild *Miscanthus* accessions out of Asia for *ex situ* research, breeding and conservation. The main tasks include planning, executing collections and quarantine, characterization in the nursery, information management and the negotiation of mutually agreed terms agreements whilst respecting the principles of the Convention on Biological Diversity (CBD).

In 2006, a European team led by IBERS travelled to China, Japan and Taiwan to collect wild *Miscanthus* germplasm. A schematic workflow for the collection is illustrated in Fig. 1. It covers planning, execution, characterization, evaluation, conservation, information management and the negotiation of agreements for the implementation of access and benefit-sharing best practice consistent with the aims and principles of the CBD.

### Planning

To ensure the success of the collection, several planning activities were carried out before the collecting trip. They include logistical planning for the materials collected to be transported to the UK in a timely and safe manner; a literature survey and use of a GPS with climate data to identify suitable locations for collecting; and preparing the quarantine facility at IBERS to accommodate the plant materials. Satellite images available through Google Maps were used extensively to identify suitable collection sites during the expedition planning. They also involved consulting with relevant authorities, such as Defra, about rhizome and seed import and export. In some cases, we needed to obtain a letter of ‘permission to enter for collection’ from the land owner beforehand.

### Germplasm collection protocols (collection model)

A comprehensive protocol was developed to handle both seed and rhizome accessions and on-site phenotyping using standardized phenotype definitions during collection. Seed-only collections will be restricted to only those plants with viable seed and can mean that vegetative high biomasses from plants with agronomically promising phenotypic traits are not represented in the collection. To this end, with assistance from the Defra Plant Health and Seeds Inspectorate (PHSI), UK, we developed a standard operating procedure (SOP) for the import of seed/panicle and vegetative rhizomatous material, under licence of a ‘Letter of Authority’ issued by Defra, into quarantine at IBERS, UK. Details of the SOP can be found in Supplementary Data S2.

At the point of collection, detailed phenotypic and site-specific environmental factors were recorded along with GPS co-ordinates. On-site phenotyping was performed during collection whenever possible. The principal agronomic traits characterized included accession type (seed/rhizome), plant height to canopy, height to top of panicle, flowering intensity, seed ripening stage, compactness of inflorescence, upright or other form of inflorescence, stem density (count) per area, secondary tillering, lodging resistance, stem to leaf ratio, stem wall thickness, stem diameter at base, stem diameter at half the canopy height, leaf breadth, radiation interception, greenness, rhizome morphology, rhizome depth, and pest and disease status.

Rhizome accessions were thoroughly cleaned of soil, and roots were trimmed to a length of <1 cm in Asia. Any rhizome material showing suspicious discoloration or damage was discarded. Cleaned rhizome pieces were wrapped in moist kitchen towel before being packaged in three layers of sealed polythene bags before shipping.

Following the CBD guidelines, all collected materials were divided into two duplicates. One duplicate set of rhizomes and seeds was shipped directly into a quarantine facility in the UK and another duplicate was kept by partners in Asia. When the rhizomes reached the UK, the packages were opened under quarantine conditions. Rhizomes were washed again, trimmed and surface sterilized using 70 % ethanol. After sterilization, rhizomes were stored in slightly damp sand which contained the insecticides Vydate (active ingredient: Oxamyl) and Tripomol 80 (active ingredient: Thiram; approx. 20 g in 40 kg of dry sand) in a cold room. After 5 months, the rhizomes were placed in pots in an isolation glasshouse under positive pressure. Plants were visually inspected in July 2007 by a UK plant health officer, and plants were subsequently moved to a lower level of quarantine in a standard glasshouse when all the risks were considered low enough. Leaf samples were sent for virus screening at the Central Science Laboratory in York, UK in April 2008, and enzyme-linked immunosorbent assay (ELISA) testing was performed. All accessions were clear of both *Miscanthus* streak virus and *Sugarcane* mosaic virus. One accession exhibited an infection with *Stagonospora* spp. Molecular work to identify the strain was carried out and the two rhizome accessions with symptoms of *Stagonospora* spp. were destroyed in September 2008. All other surviving rhizome accessions were cleared for release in October 2008 following a Defra plant health inspection, and were split and moved from quarantine to field nurseries.

Seed accessions (some on panicles) were ripened under quarantine, and the panicles were then threshed. Clean seed was surface sterilized using approx. 10 % sodium hypochlorite (full-strength Domestos, Unilever, London, UK) for 1 h before germination on water agar or MS (Murashige and Skoog) medium under constant conditions of 25 °C with 100 μmol m^–2^ s^–1^ of light. After 30 d, where no fungal or bacterial contamination was observed on the media, seedlings were transferred to modular trays and raised in a glasshouse.

### Field observation nursery and replicated trial establishment

The field nursery was established on a sloping field (52°26′N, 04°01′W) near Aberystwyth on the west coast of Wales, exposed to winds from the south and west. The soil is classified as a dystric cambisol (FAO, 1988) with a stone fraction (particles >2 mm) of approx. 50 % of the soil mass in the 0–40 cm layer. Climate data (rainfall, temperature and radiation) were obtained from the Gogerddan (Aberystwyth) weather station (52°25’N, 04°01’W). Lateral rhizome growth of plants which were planted in the field was restricted by galvanized steel collars inserted into the ground (0.56 m diameter × 0.2 m deep × 1.5 mm thick) and have proven effective in keeping accessions separated for >10 years.

### *Ex situ* field phenotyping of wild *Miscanthus*

A total of 100 rhizome or seed accessions were selected from the collection and planted in 2009 in a field observation nursery at Aberystwyth, UK for characterization, evaluation and conservation, and as a supply of clones for use in research and breeding. The 100 accessions comprised 16 *M. sinensis*, 34 tetraploid *M. sacchariflorus*, 11 diploid *M. sacchariflorus* from northern China, 36 ssp. *lutarioriparius*, two *M. floridulus* and one accession of ssp. *transmorrisonensis*. Following establishment in 2009, phenotyping of plants was undertaken in year 2 (Y2) (2010), Y4 (2012) and Y5 (2013). At the end of the growing season (October), a number of biomass component traits were measured including canopy height, basal diameter (the diameter of the clump at ground level), stem transect count (the number of stems counted along a transect inserted through the middle of the clump at approximately half canopy height), stem diameter (the average of three stem diameters taken at mid-internode at approximately half canopy height) and tallest stem (the height to the highest part of the stem, excluding the flower, if present). Details of the plant phenotyping protocols were described by Jensen *et al.* (2011*a*) and Robson *et al.* (2013*a*). Dry matter yield (DMY) per plant was not measured during the establishment year but biomass was removed after senescence. The plants were subsequently harvested in February 2011, 2013, 2014 and 2016, and DMY was measured.

A selection of 101 accessions (26 *M. sinensis*, 34 tetraploid *M. sacchariflorus*, 11 diploid *M. sacchariflous* from northern China, 29 ssp. *lutarioriparius* and one accession of ssp. *transmorrisonensis*) were included as a sub-set in a 1000 plant, triply replicated (3 × 1000) association mapping trial in Aberystwyth, UK. The trial was established in 2012. The phenotyping was performed, and DMY was measured following the third and fourth growth years in spring 2015 and 2016, respectively.

### Harvesting method, dry matter yield (DMY) and moisture content (MC%) calculation

Plants were harvested in the early spring. Plants are typically harvested in the UK and Europe after the winter when biomass quality is improved in terms of reduced moisture and nutrient content, despite a concurrent loss of biomass yield relative to peak yield (Clifton-Brown *et al.*, 2008). Whole plants were harvested at a height of approx. 5 cm from the soil surface and chopped to 10 cm lengths. The cut biomass was collected in a plastic bag and weighed to determine total fresh weight (f. wt total). A sub-sample of approx. 200 g was removed and weighed to determine the sub-sample fresh weight (f. wt sub-sample). The sub-sample was then dried to a constant weight at 60 °C before reweighing (d. wt sub-sample). The percentage moisture content (MC%) was then calculated using the formula MC% = 100 × (f. wt total – d. wt total)/f. wt total. The whole plant dry weight was estimated: d. wt total = f. wt total × (d. wt sub-sample/f. wt sub-sample).

### Ploidy test methodology

A 2 cm piece of fresh leaf was chopped in 2 mL of ice-cold buffer (0.5 m citric acid monohydrate, 0.5 % Tween-20) with a razor blade to release cell nuclei. The suspension of cell nuclei was then stained by adding 0.5 mL of 0.4 m anhydrous Na_2_HPO_4_ containing 4 mg mL^–1^ DAPI (4′,6-diamidino-2-phenylindole), filtered through a 50 mm nylon mesh and run on a ploidy analyser (http://www.partec.com). Histogram peaks representing relative DNA content were recorded and compared with known diploid *M. sinensis* and a tetraploid *M. sacchariflorus* by chromosome counting under a microscope.

### Genebank and technologies for germplasm evaluation and breeding

Passport data for this collection have been submitted to and are managed by the Aberystwyth Genetic Resources Information System (AberGRIS) which is mirrored in the UK National Plant Inventory and the European Search Catalogue for Plant Genetic Resources (EURISCO). The IBERS genebank is managed following the procedures and guidelines laid down by IPGRI Handbooks for Genebanks No. 6. A guide to effective management of germplasm collections (Engels and Visser, 2003); IPGRI Handbooks for Genebanks No. 7. Technical guidelines for the management of field and *in vitro* germplasm collections (Engelmann and Engels, 2002; Reed *et al.*, 2004); CBD Article 8 and 9 for *ex situ* conservation; and ‘Genebank standards for plant genetic resources for food and agriculture’ (FAO, 2014).

**Fig. 2.**
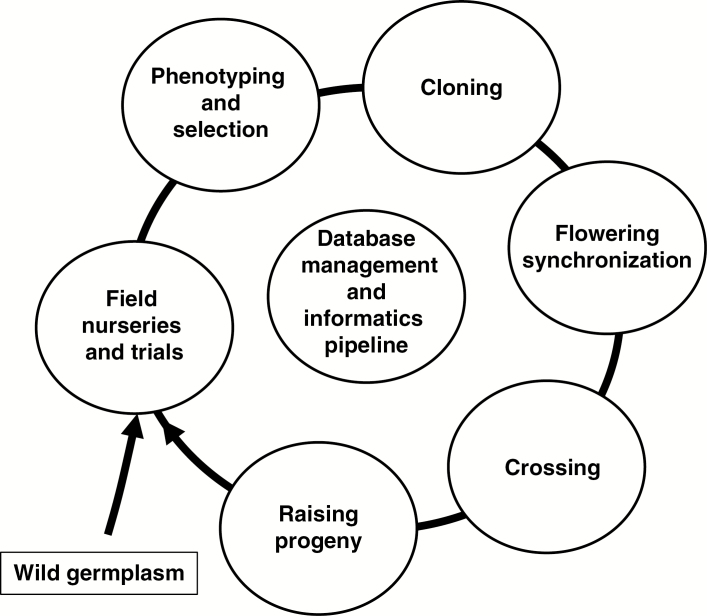
The breeding cycle showing the entry point for the exploitation of novel wild germplasm and the subsequent steps: phenotyping and selection, cloning, flowering synchronization, crossing and raising seedling progeny. The informatics pipeline plays a central role in managing, analysing and visualizing information generated within and between steps.

A major purpose of the collection was to increase the genetic diversity and quality of *Miscanthus* in the UK and Europe available for plant breeding. To initiate this, a cyclical germplasm improvement scheme, including selection, flowering synchronization, crossing and evaluation, was established, as illustrated in Fig. 2.

A versatile data management and informatics platform, MScan, was established based on Ruby-on-Rails (http://rubyonrails.org) and several informatics technologies to manage and assist in the germplasm evaluation, improvements and all stages of breeding. The technologies used and their roles in building this platform are listed in Table 1. An easy to use tablet-based phenotyping tool, using Android Java API development (https://developer.android.com/guide/index.html) technology with the SQLite (https://www.sqlite.org/index.html) database to store phenotype data in the tablet, and JSON (https://www.json.org) to communicate with MScan, was also built to facilitate phenotyping.

**Table 1.** A list of novel informatics technologies used to build a versatile data management and informatics platform, MScan, to manage and assist in the germplasm evaluation, improvement and all stages of breeding

Name	Role	Link/reference
Linux	Linux has been used as the main server’s operation system running on a high-performance 6-core server with 16 Gb RAM	https://www.linux.org
Ruby-on-Rails	Ruby-on-Rails is an open-source, object-oriented web development environment. It was used to develop the database system with Ruby as the programming language	http://rubyonrails.org
MySQL	MySQL, an open-source relational database management system, has been used as MScan’s underlying database.	https://www.mysql.com
jQUERY	jQUERY, a cross-platform JavaScript library, was used to underpin effective query in database using JSON.	https://jquery.com https://www.json.org
Nginx	Nginx was used to establish a high-performance web server for MScan	https://nginx.org/en/
Mongrel	Mongrel, an open-source software HTTP library and web server, has been used as a load balancer for the Rails application	https://rubygems.org/gems/mongrel
Subversion	Subversion was built-in as the Linux source code control system	https://subversion.apache.org

MScan also performs the function of genebank information management. For example, each rhizome was assigned an ‘accession no.’ by MScan when it entered the collection at IBERS. Clones divided from each accession were planted for use in various fields, grasshouses and locations. Each clone is assigned an unique identifier (UID) and its current status and family history can be tracked using MScan.

### Statistical analysis and modelling

The statistical package R (R Core Team, 2013) was used to conduct both linear model and RandomForest model analyses. The linear model analysis was performed using the distance-based linear model method, and the RandomForest model analysis was performed using the RandomForest library (Liaw and Wiener 2002). For both models, five parameters were used, i.e. species, stem count, stem diameter, tallest stem height and the flowering score. The RandomForest model incorporated these five attributes/parameters to create multiple training data sets and trees to predict importance scores. Predictions were taken as the average of all the trees,

$$F(x^{\prime })=\frac{1}{B}\sum_{b=1}^{B}f(x^{\prime })$$

where *B* is the number of trees.

## RESULTS

### Novel germplasm collection

**Fig. 3.**
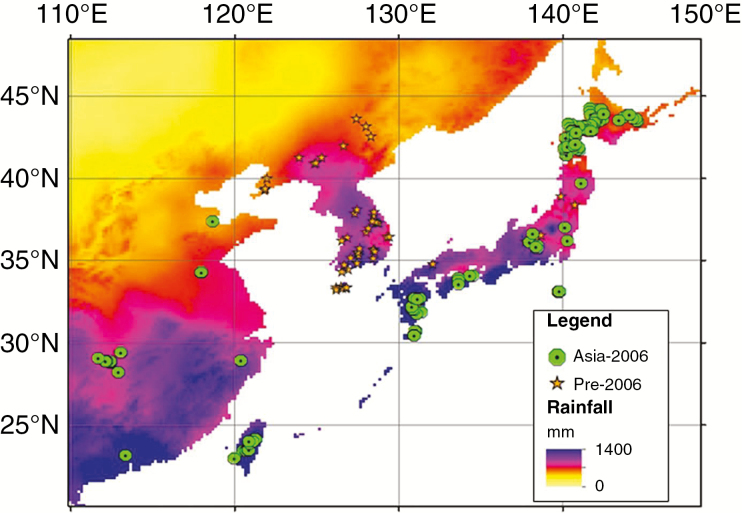
One hundred and fifty-eight site locations collected from Asia in 2006 plotted with 58 sites collected by Kew and ADAS between 1997 and 2001. The Asia-2006 collection sites in Eastern Asia range from 23° to 44°N, 111° to 144°E and altitudes from –3 m to 3123 m with a wide climatic range which is here represented by annual rainfall. Annual rainfall (in mm) is shown using a colour scale, with deep blue representing high annual rainfall (>1400 mm) and yellow representing annual rainfall approaching 0.

In 2006, a total of 303 *Miscanthus* accessions in the form of both seed and rhizomes were collected from 158 locations between September and December in South-east Asia. Species collected included 133 *M. sinensis*, 137 *M. sacchariflorus* (including ssp. *lutarioriparius*), 28 *M. floridulus*, two *M. sinensis* ssp. *transmorrisonensis* and three interspecific hybrids. The 158 collection sites ranged in latitude between 23° and 44°, longitude 111° and 144° and altitude –3 m and 3123 m. The GPS co-ordinates of 158 sites of the Asia-2006 collection and 58 known collection sites of pre-2006 collections were anchored onto a rainfall (in mm) spatial map as illustrated in Fig. 3. Accessions were collected from diverse environmental conditions including high-salinity soil, a volcanic sulphur mine, small Pacific islands in the northern part of the Ryukyu Islands in Japan and land that experiences seasonal flooding. Site information, including latitudes, longitudes and altitudes, countries of collection, species and ploidy level is summarized in Supplementary Data Table S3.

**Fig. 4.**
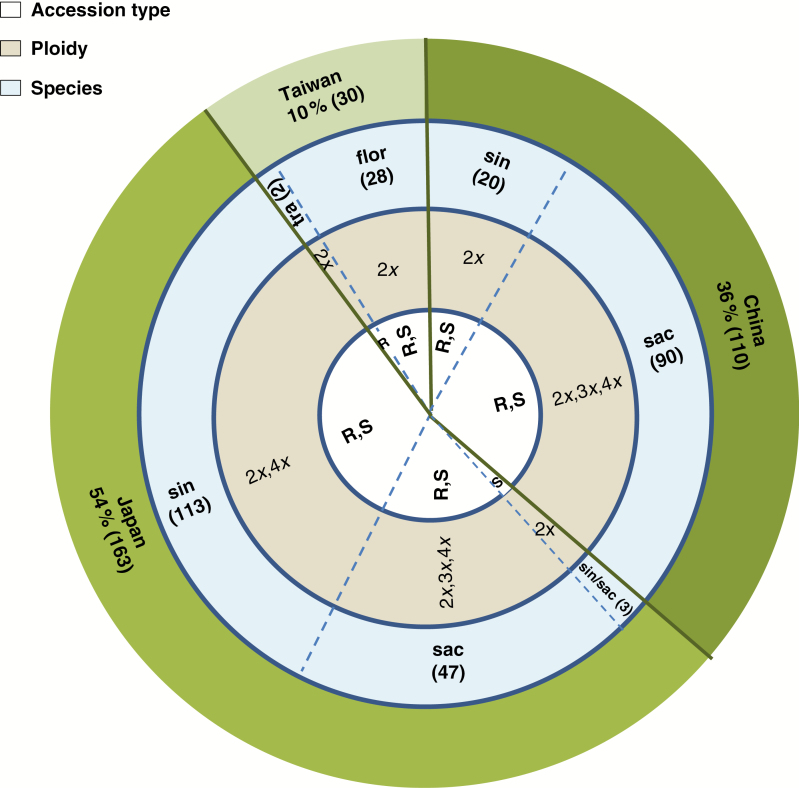
A visual summary of the Asia-2006 collections (303 accessions) showing country of origin (China, Japan and Taiwan), species type, ploidy level (2*x*, 3*x*, 4*x*) and accession type (Rhizome or Seed). The species include *M. sinensis* (sin), *M. sacchariflorus* and ssp. *lutarioriparius* (sac), *M. floridulus* (flor), the natural hybrid between *M. sinensis* and *M. sacchariflorus* (sin/sac) and *M. sinensis* ssp. *transmorrisonensis* (tra).

The ploidy level was measured in surviving accessions. The Asia-2006 collection, sorted by country of origin (China, Japan and Taiwan), species, ploidy level and accession type is summarized in Fig. 4.

#### China collection.

The majority of the 110 accessions collected from China (36 % of the total collection) comprised *M. sinensis*, *M. sacchariflorus* and ssp. *lutarioriparius* genotypes. Most of the rhizome material survived shipping despite spending several weeks in transit. Most seed accessions carried at least some viable seeds.

#### Japan collection.

A total of 163 accessions were collected in Japan (54 % of the total collection) and they comprised mostly *M. sacchariflorus* and *M. sinensis*, and in particular diploid *M. sinensis* and tetraploid *M. sacchariflorus*, the parental species of the triploid natural hybrid, *M. × giganteus*.

#### Taiwan collection.

A total of 30 *M. floridulus* and *M. sinensis* ssp. *transmorrisonensis* accessions (10 % of the total collection) were collected in the mountainous areas of central Taiwan. Many plants were not flowering at the time of collection, and therefore most accessions were collected as rhizomes.

### Implementation of the Convention on Biological Diversity (CBD) and access and benefit-sharing best practice

We worked closely with Defra and international partners in Asia to develop bi-lateral agreements containing mutually agreed terms (MATs) consistent with the framework of the CBD. Agreements signed with Chinese, Japanese and Taiwanese institutions covered access to the specifically collected and defined germplasm for scientific evaluation and exploitation.

The agreements embodied appropriate care, best collection practice with good record tracking systems and respect for individual community and national laws and procedures as well as the customary practices of use and exchange. For example, we took into consideration: (1) the national legislation status of each individual country with respect to the progress in implementing the CBD and the Nagoya Protocol; and (2) the need for land owners’ rights to be respected in Japan when obtaining permission to collect. The terms and conditions of these agreements therefore vary in order to address the individual needs and requests of the other parties. Several generic terms form the basis of these agreements including (1) this germplasm collection is a joint research effort between donor and recipient institutions; (2) allow access for collection; (3) allow transfer of materials to a recipient country for research; (4) produce joint publications; and (5) pursuit of joint research grant opportunities and knowledge exchange.

We also actively engaged in activities to implement other CBD Articles. They include (1) promoting international collaboration through joint research efforts and publications (Articles 5, 10, 12 and 13); (2) developing a proper germplasm and data management, genebank, tracking and analysis system for appropriate monitoring and maintenance (Article 7); (3) providing incentive measures by appropriate exchange and transfer of relevant technologies and expertise (Articles 11, 16, 17 and 18); (4) *ex situ* conservation (Articles 8 and 9); and (5) ensuring the fair and equitable sharing of the benefits arising out of the utilization of genetic resources (Articles 15, 19, 20 and 21). Examples of and evidence for these implementation activities can be found in Table 2. A detailed description of activities of the steps we took to achieve compliance with CBD Articles and their potential contribution and impact areas for this collection are also included in Table 2.

**Table 2.** A summary of the implementation measures achieved by the Asia-2006 *Miscanthus* germplasm collection, associated CBD Articles, potential contribution and impact areas to achieve compliance with CBD and benefit sharing to institutions in countries where *Miscanthus* was collected

Implementation Measures	Relevant CBD Articles	Evidence of compliance with CBD in making this collection	Contribution and impact areas
Respect jurisdiction, national law and practice of individual community when accessing genetic resources	Article 4 and 10	Worked with Hokkaido University in Japan to obtain the permission and consent of landowners of our collecting sites for access and the utilisation of materials collected.	Conservation and ethical and sustainable use of genetic resources
Actively engage in international cooperation	Article 5 and 10	Research MOUs for collaboration have been signed between Aberystwyth University and partners in Asia. IBERS has been awarded (with partners in Asia) a BBSRC international partnering award (BB/L003953/1) and BBSRC international workshops (BB/M027643/1) on bioenergy R&D to exploit the materials collected.	R&D on bioenergy; conservation; and ethical and sustainable use of genetic resources
Develop a proper data management and analysis system for monitoring, identification and exploitation of data collected	Article 6	A custom-designed and developed relational database and visualization system (MScan) was established to manage, track and query collection data sets and traditional knowledge. Data from field trials were analysed to inform breeding programmes.	R&D on bioenergy; conservation; ethical and sustainable use of genetic resources
Provide incentive measures such as joint research efforts and publications	Article 7	Joint research grant applications have been submitted with partners in Asia to the UK Newton Fund and Global Challenge Research Fund Initiatives. Several joint publications with partners in Asia: Clifton-Brown *et al.*, 2008, 2011, 2016; Toma *et al*., 2013.	R&D on bioenergy and conservation
Increase diversity of *Miscanthus* germplasm collection in Europe	Article 7 and 9	The collection has expanded and enriched the genetic diversity of *Miscanthus* germplasm collections in Europe as illustrated in this publication.	R&D on bioenergy and adaptation
Conduct *Miscanthus* breeding and research to produce environmentally adaptable and resilient varieties	Article 12 and 13	IBERS has been awarded funding for several *Miscanthus* germplasm improvement programmes by Defra, EU, BBSRC and UK regional government including GIANT (Defra/BBSRC LK0863), BBSRC Institute Strategic Programme grant (BBSEG00003134) and EU projects to exploit and produce diverse varieties from the materials collected. One of our EU FP7 projects OPTIMISC has performed life cycle analysis to identify optimum production scenarios and maximize the environmental, economic and social benefits of growing *Miscanthus* in Europe and China.	R&D on bioenergy; conservation and adaptation
Establish mechanisms and agreements for the fair and equitable sharing of benefits arising from utilization	Article 15	Contractual ‘mutually agreed terms (MAT)’ agreements have been signed between Aberystwyth University and each individual partner in Asia to obtain access and agree the terms of future benefit sharing arising from the utilization of collected materials.	R&D on bioenergy; conservation and ethical and sustainable use of genetic resources
Encourage exchange and transfer of relevant technologies and expertise with partners	Article 16, 17 and 18	Three international bioenergy workshops have been held in Taiwan in 2009, 2012 (green chemistry and bioenergy) and 2015 (bioenergy and innovation) attended by partners in Asia and Europe to exchange and share knowledge, technologies and expertise. Students and staff from our Asian partners visited IBERS in 2016 and 2018.	R&D on bioenergy; conservation and ethical and sustainable use of genetic resources
Encourage *in situ* conservation in their native environments and conduct *ex situ* conservation in the UK	Article 8 and 9	Provide incentives through joint research projects to encourage our partners in Asia to perfornm *in situ* conservation. Establish the seed bank (seed) and field genebank (rhizome) at IBERS by adopting the proper protocols and guidelines laid down by IPGRI and FAO for management of genebanks (Engelmann and Engels, 2002; Engels and Visser, 2003; Reed *et al.*, 2004; FAO 2014). Passport data of collected materials have been deposited into Aberystwyth Genetic Resources Information System (AberGRIS) which is mirrored in UK National Plant Inventory and EURISCO. Accessions can be accessed via UKNPI URL: https://fusiontables.google.com/DataSource?docid=1e1Eb9ZN8-MKg9f8XZnDnaNaO-d8nQzfbxmqYt3zg#chartnew:id=4 The accessions numbers of this ‘Collected Materials’ in AberGRIS are: M06A1–M06A305. A versatile data management and informatics platform, MScan, has been established to manage and tracking genebank information for research and breeding at IBERS.	Conservation and ethical and sustainable use of genetic resources

### Exemplary field nurseries and observations on yield performance of selected genotypes that illustrate diversity collected

**Fig. 5.**
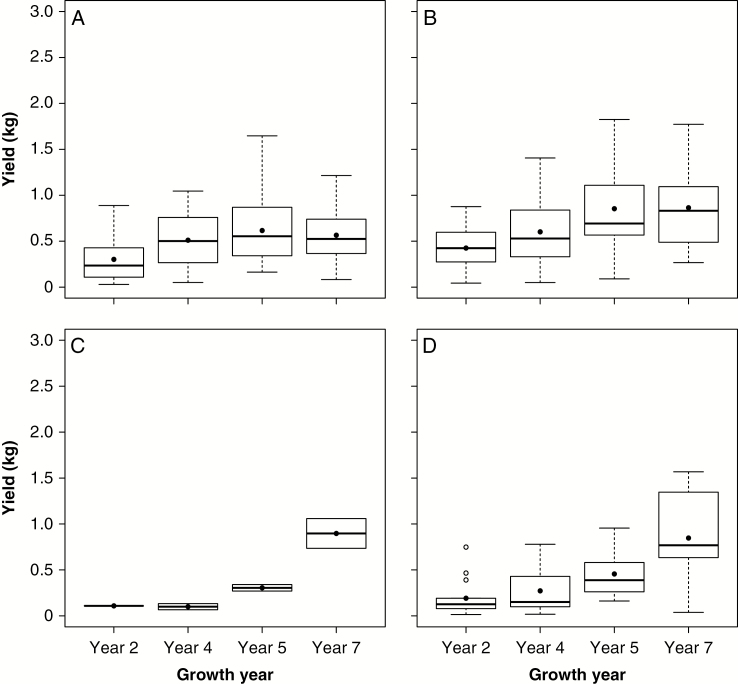
Spring harvestable yield (kg dry matter per plant) from Chinese *M. sacchariflorus* (A), Japanese *M. sacchariflorus* (B), Taiwanese *M. floridulus* (C) and *M. sinensis* collected from all three countries (D) grown in a spaced plant nursery trial at Aberystwyth, UK. In box plots, outliers are represented as circles, the box illustrates upper and lower quartiles, the solid line within each box represents the median, and the filled circle denotes the mean.

The 100 plants selected and grown in field nurseries in Aberystwyth, UK were phenotyped, harvested (in February 2011, 2013, 2014 and 2016) and their DMY measured. The *ex situ* productivity (in terms of spring harvestable above-ground biomass) of the 100 Asia-2006 accessions showed that *M. sacchariflorus* from China (Fig. 5A) and Japan (Fig. 5B) reached full productive potential by the fifth growing season. The productivity of the low-latitude Taiwanese *M. floridulus* (Fig. 5C) was initially low for the first 5 years, but by the seventh growing season these accessions were similarly productive as the *M. sacchariflorus* genotypes collected from high latitudes in China and Japan. As with *M. floridulus*, early yields from *M. sinensis* genotypes (collected from all countries) were less than those of *M. sacchariflorus*. However, in later harvests, yields of *M. sinensis* reached similar levels (Fig. 5D). By 7 years after planting, only the *M. sacchariflorus* types had reached peak yield and apparently stabilized their yields (Fig. 5A, B) while those of *M. sin*ensis and *M. floridulus* plants were still increasing year on year.

**Fig. 6.**
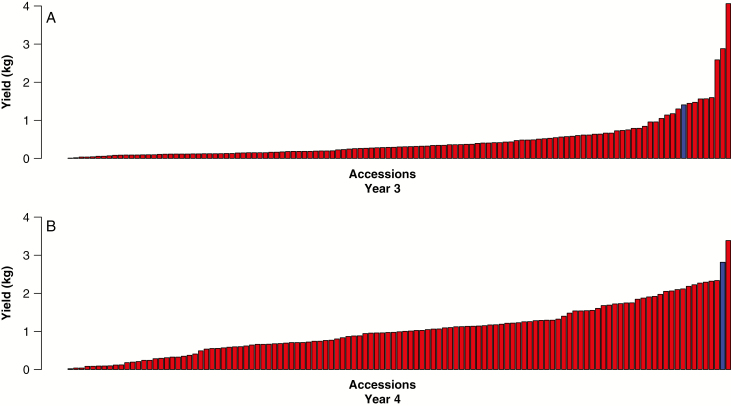
Bar charts showing dry matter yield (DMY) of 101 accessions from the Asia-2006 collection as part of a 1000-plant three times replicated association mapping trial based at Aberystwyth, UK in year 3 (A) and year 4 (B). Red bars are the Asia-2006 accessions and the blue bar denotes the control *M.* × *giganteus* grown in the same trial.

A total of 101 accessions were selected from the collection and included in a three times replicated association mapping trial of 1000 accessions. The DMYs for these 101 accessions in year 3 and year 4 (Fig. 6A, B) indicate that there is considerable diversity within the collections for yield potential in the UK and Europe.

**Fig. 7.**
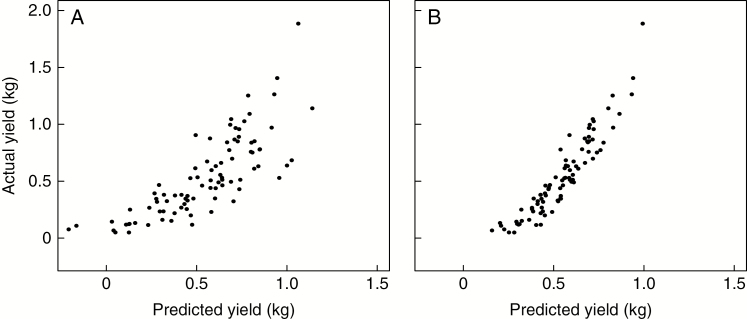
Regression of modelled yield from a linear model (A) and a RandomForest model (B) using input traits (species × stem count × stem diameter × flowering score × tallest stem) and observed spring harvest yields in a 1000-plant three times replicated association mapping trial at Aberystwyth, UK. The success of this analysis is measured by how close the ‘predicted yields’ are to the ‘actual yield’. The RandonForest model predicts actual yield better than the linear model in test data.

Linear and RandomForest modelling were used to analyse the predicted vs. actual DMY (Fig. 7A and B, respectively). RandomForest modelling was also used to determine the importance of the phenotype in contributing to the yield. The importance scores generated from the RandomForest model for each characteristic that contributed to the DMY measured in early 2013 (following the 2012 growing season) were (1) species, 0.8150290; (2) stem count, 2.1629454; (3) stem diameter, 0.9074870; (4) tallest stem, 2.0440019; and (5) flowering time, 0.2361554.

### An assessment of the potential for making useful F_1_ hybrids

**Fig. 8.**
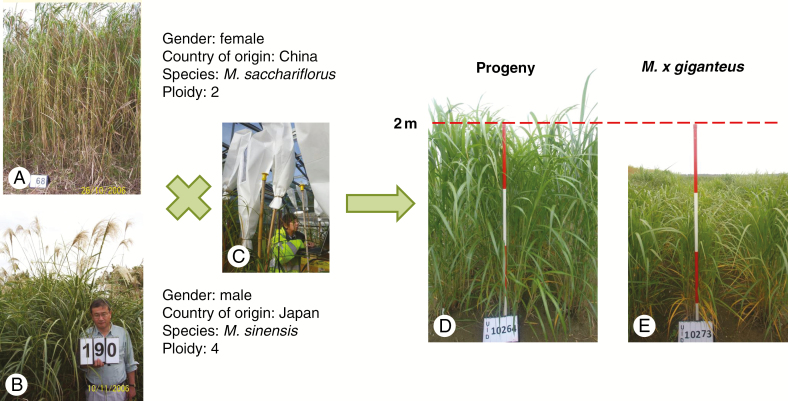
An example of an F_1_ wide cross between Chinese diploid *M. sacchariflorus* and Japanese tetraploid *M. sinensis* both collected in 2006. The female (A) and male (B) parents were crossed in the glasshouse (C) and produced progeny which were planted in a replicated trial (3 × 2 m per plot and two plants per m^2^) in May 2012 incorporating *M. × giganteus* as control. Both the progeny (D) and *M*. × *giganteus* (E) were photographed in October 2012.

**Fig. 9.**
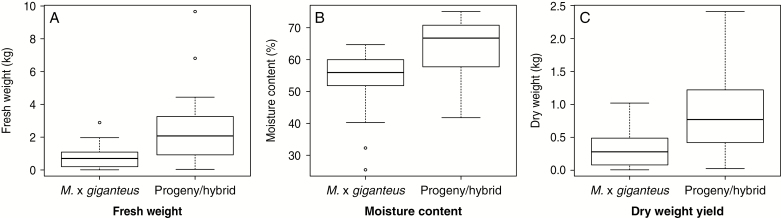
Box plots comparing control *M.*×*giganteus* and an F_1_ progeny/hybrid for fresh weight (A), moisture content (B) and dry weight yield (C) per plant of the second-year growth.

**Fig. 10.**
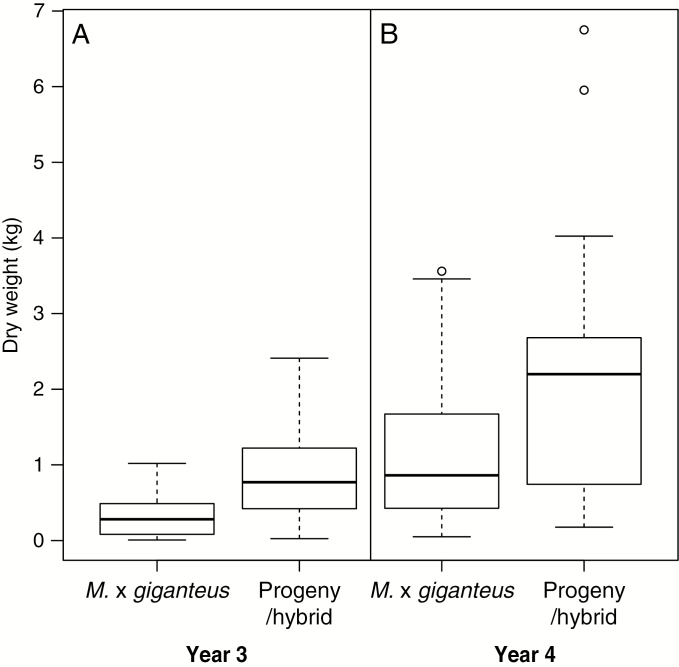
Box plots comparing control *M*.×*giganteus* and an F_1_ progeny/hybrid for the dry weight yield of the third year (A) and fourth year growth (B).

Over 600 crosses were made between fertile accessions collected in 2006, and >180 of these crosses were successful in producing seed. Progeny were planted out in various types of trials to assess the biomass potential relative to *M. × giganteus*. An example of an F_1_ hybrid cross involving accessions collected in 2006 that produced seed is illustrated in Fig. 8. The parents of the cross were a Chinese *M. sacchariflorus* (2*x*) and a Japanese *M. sinensis* (4*x*). In a triplicate mapping population trial, the progeny of this cross exhibited an improved first year establishment (in terms of autumn canopy height, stem count and yield measured in spring) compared with *M*. × *giganteus*, and the improved yield performance was also observed in year 2 (Fig. 9A–C), year 3 (Fig. 10A) and year 4 (Fig. 10B).

## DISCUSSION

The transfer of *Miscanthus* germplasm from its countries of origin in Asia was needed to facilitate plant breeding. A comprehensive science-based system for the generation, curation and exploitation of a collection of *Miscanthus* accessions (Fig. 1) was established which followed the principles and guidelines of the CBD including the making of agreements with institutions in each country from which germplasm originated. These agreements enabled access to the wild *Miscanthus* germplasm in Asia and their transfer to Europe for research and exploitation. They also pave the way for other institutions to request the access of live materials of this collection via appropriate contractual arrangements with AU and other relevant parties. Any third party wishing to access the ‘collected materials’ will require a bilateral agreement with AU that respects and takes into consideration the terms of the CBD agreements that AU has signed with three Asian partners. For example, for research purposes, the third parties can request access to collected materials through a research-only material transfer agreement (MTA) with AU. Breeding and subsequent utilization with the potential for commercialization necessitates a separate ABS agreement containing the terms of benefit sharing following the best practice of the Nagoya Protocol and 2014 EU Regulation No 511/2014. That the nation state has sovereignty rights over indigenous germplasm is one of the most important elements of the CBD (Schroeder and Pisupati, 2010). Nation states are therefore to be rewarded where access is granted and commercial returns are made from their germplasm resources. Benefit can be in monetary form (royalty payment, licence fee, etc.) or a non-monetary benefit sharing, and can bring both economic returns and technological advances to developing countries (Roa *et al.*, 2016). Furthermore, according to Winge (2014), the CBD and the practice of access and benefit sharing can contribute to a more resilient agriculture under the conditions of climate change.

As illustrated in Table 2, non-monetary benefit sharing includes joint research efforts and publications, knowledge transfer, joint post-graduate student exchange, sharing research results and access to state-of-the-art facilities, such as the UK National Plant Phenomics Centre based at IBERS, which have been shown to have an impact on technological advances in *Miscanthus* research by our Asia partners. In order to ensure that the donor institution/country receives a fair and reasonable share of the benefits gained by the recipient of collected materials as a result of the commercial exploitation of the collected materials by the recipient (or third party), the monetary benefits, such as royalties, received by the recipient will be shared with the donor institution/country. The royalty payment structure is proportional to the commercial sale value of the plant materials derived from the collected materials. The royalty rates take account of those for existing commercialized crops and are mutually agreed between donor and recipient.

The International Treaty on Plant Genetic Resources for Food and Agriculture (ITPGRFA) for the 64 most important crops (Annex I crops), which together account for 80 % of all human consumption (Plant Treaty, 2014), has established a multilateral system for benefits sharing. This requires recipients of plant materials to pay an equitable share of the financial benefit they gain into ITPGRFA’s Benefit-sharing Fund to fund farmers in developing countries for conservation and sustainable use of plant genetic resources. However, the use of non-Annex I crops, such as *Miscanthus*, for benefit sharing is based on a bilateral system. Currently no similar multilateral system benefit-sharing fund for non-Annex I crops for benefit sharing has yet been established. Implementing benefit collection therefore relies on the donor institution/country setting up a fund for receiving monetary benefits. The fund is, in general, intended to allow flexibility and is not limited to funding farmers and land owners to conserve diversity. It is also available for further research on diversity conservation and sustainable use of genetic resources by institutions in the donor countries. Potential monetary and non-monetary benefits accruing from access and benefit sharing are listed in the Bonn guidelines (United Nations, 2002).

The outcome of Asia-2006 is a diverse and ethically accessed collection of Asian wild *Miscanthus* germplasm for use in Europe for breeding to help in diversifying agriculture (Donnison and Fraser, 2016), climate change mitigation and for the long-term *ex situ* conservation of an important grass species.

Based on the summary information presented in Figs 3 and 4, there is strong evidence that the collection comprises a high level of diversity with coverage that spans species, ploidy, countries of origin and environmental conditions at the site of collection. This collection therefore provides additional genetic diversity which can be used to help in meeting breeding targets such as resilience to abiotic stresses and the ability to perform well in a wide range of climates and environments. Considerable genetic diversity was also identified in biomass yield performance within the collection (Fig. 6).

The analysis of 7 years of observations on selected genotypes planted in a field observation nursery has confirmed that *M. floridulus* species required a longer establishment period than other species (Fig. 5C). Yield is a complex of traits, with no single trait contributing more than 33 % of DMY. Collectively the tallest stem, stem count and stem diameter measurements predicted approx. 60 % of yield contribution; hence, these three traits can account for the majority of heritable yield (Robson *et al.*, 2013*b*). RandomForest modelling (Fig. 7B) suggested and confirmed that the combination of the three traits (tallest stem, stem count and stem diameter) could explain >60 % of DMY.

That an F_1_ hybrid from a wide cross (Fig. 8D) between a Chinese *M. sacchariflorus* and a Japanese *M. sinensis* was able to outperform *M. × giganteus* during establishment (Fig. 8D, E) and in subsequent years of growth performance (Figs 9C and Fig. 10A, B) indicates the potential of the collection for crop improvement. The identification of such a hybrid is commercially relevant because a rapid establishment of a perennial is important to early economic returns for growers of the crop and otherwise can present a barrier to adoption. Heterosis can be displayed by the progeny of genetically diverse parents including those that belong to different species. These results suggest the potential of making new wide crosses between plants of different species, ploidy levels and countries as a route to replicate and improve on the performance of the naturally occurring, and highly productive hybrid, *M*. × *giganteus*. The challenge in breeding perennial grasses, however, is to ensure that performance is maintained over the life cycle of the crop, which for *Miscanthus* may be 10–20 years, and across multiple environments.

Within the collection from Japan, there was one triploid accession, based on assayed nuclear DNA content, where diploid *M. sinensis* and tetraploid *M. sacchariflorus* were growing in close proximity. A number of studies have indicated that interspecific triploid hybrids similar to *M.* × *giganteus* can potentially release significant heterosis and produce higher yields at maturity (Chae *et al.*, 2013; Robson *et al.*, 2013*b*; Clark *et al.*, 2015). The discovery of triploids in the wild justifies the strategies of collecting diploid *M. sinensis* and tetraploid *M. sacchariflorus* for breeding and of identifying possible sites to collect triploid accessions from Japan that we adopted before the collection. The ability to recreate such hybrids, and therefore genetic diversity in the crop, is also highly important to increase the resilience of the industry to any pests and diseases that may emerge.

Conservation of diversity is one of the paramount priorities of the CBD. *Miscanthus × giganteus* is a sterile clone which has limited value for conservation apart from any epigenetic diversity that has appeared during somatic propagation. The selection of new and fertile wild germplasm in Asia and its transfer to the UK described here has increased the number of places where *ex situ* conservation of *Miscanthus* germplasm is being undertaken. This is particularly important for a previously undomesticated wild species such as *Miscanthus* which is at risk of loss of diversity as some areas are cleared for other land uses. The living collections from Asia-2006 planted in the UK nursery trials perform the roles of both a genebank and a reservoir for breeding selections.

The collection has added to the existing *Miscanthus* genetic resources and will in the future be further characterized for important traits as has occurred in populations for flowering time (Jensen *et al.*, 2011*a*), drought resistance (Malinowska *et al.*, 2016), salinity tolerance (Stavridou *et al.*, 2016) and resistance to low temperatures (Fonteyne *et al.*, 2016). Such traits are all keys to tailoring hybrids to make the most efficient use of the growing season and extend the productive range of the crop on marginal lands (McCalmont *et al.*, 2017). Knowledge of these traits should enable the breeding of new varieties based on hybridizations that would not occur readily in nature due to barriers of geography and asynchronous flowering time. Moreover, if hybrid seed production can be induced to occur at sufficiently high efficiency and low cost between parents that give rise to high-yielding hybrids, then a seed-based scalable industry, which is much more cost effective than a clonal one based on *M.* × *giganteus* (Clifton-Brown *et al.*, 2016), can become a reality and one in which rewards can be justly returned to the countries where the parental germplasm originated.

## SUPPLEMENTARY DATA

Supplementary data are available online at https://academic.oup.com/aob and consist of the following. S1: further descriptions of the 1993 Convention for Biological diversity (CBD), 2010 Nagoya Protocol and 2014 Regulation (EU) No 511/2014 on compliance measures for users from the Nagoya protocol on access to genetic resources and the fair and equitable sharing of benefits arising from their utilization. S2: standard operating procedure (SOP) for the import of vegetative rhizomatous material into quarantine in the UK under licence from Defra Plant Health and Seeds Inspectorate (PHSI). Table S3: summary of site information including latitudes, longitudes and altitudes, species, ploidy levels and countries of collection of 158 collection sites where 303 accessions of germplasm were collected between September and December 2006.

mcy231_suppl_Supplementary_Figure_1

mcy231_suppl_Supplementary_Figure_2

mcy231_suppl_Supplementary_Figure_3

mcy231_suppl_Supplementary_Figure_4

mcy231_suppl_Supplementary_Figure_5

mcy231_suppl_Supplementary_Figure_6

mcy231_suppl_Supplementary_Material_S1

mcy231_suppl_Supplementary_Material_S2

mcy231_suppl_Supplementary_Material_S3
