# Supplementary figures and images for: Collecting wild Miscanthus germplasm in Asia for crop improvement and conservation in Europe whilst adhering to the guidelines of the United Nations’ Convention on Biological Diversity
Source: Ann Bot. 2018 Dec 22;124(4):591–604. doi: 10.1093/aob/mcy231 (PMC6821356; doi:10.1093/aob/mcy231)

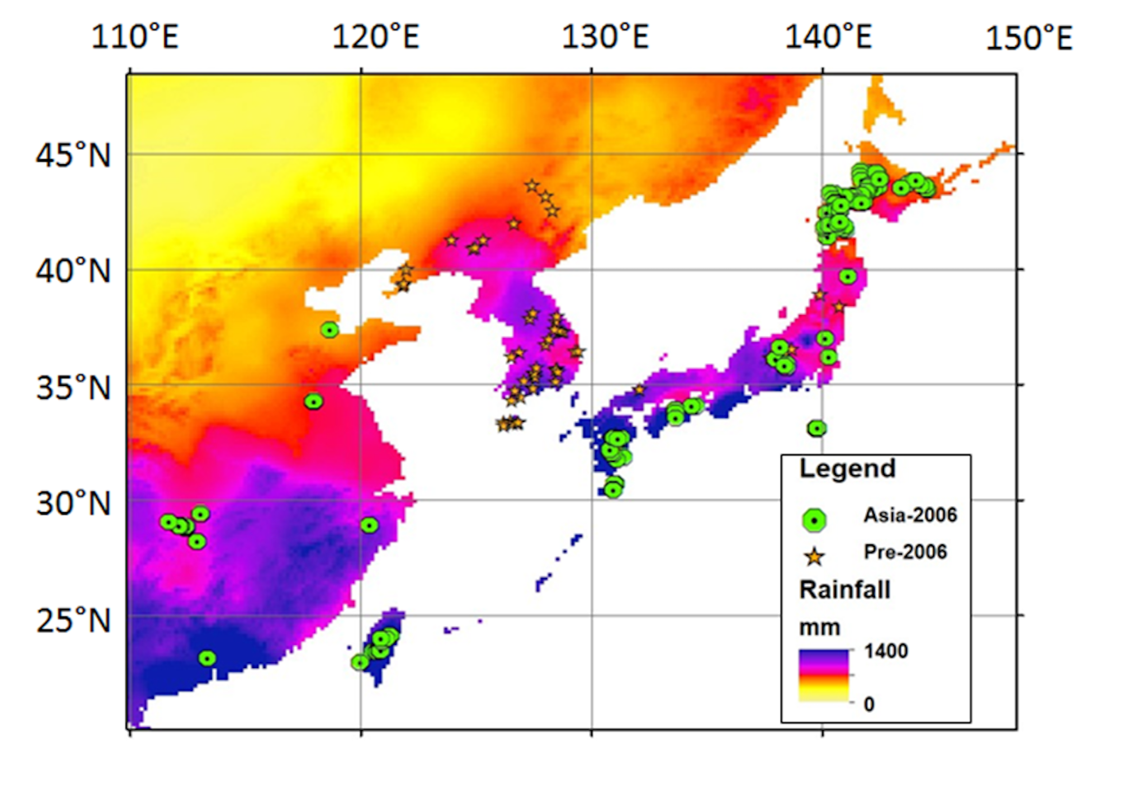

Supplement: mcy231_suppl_Supplementary_Figure_1 [file mcy231_suppl_supplementary_figure_1.png]

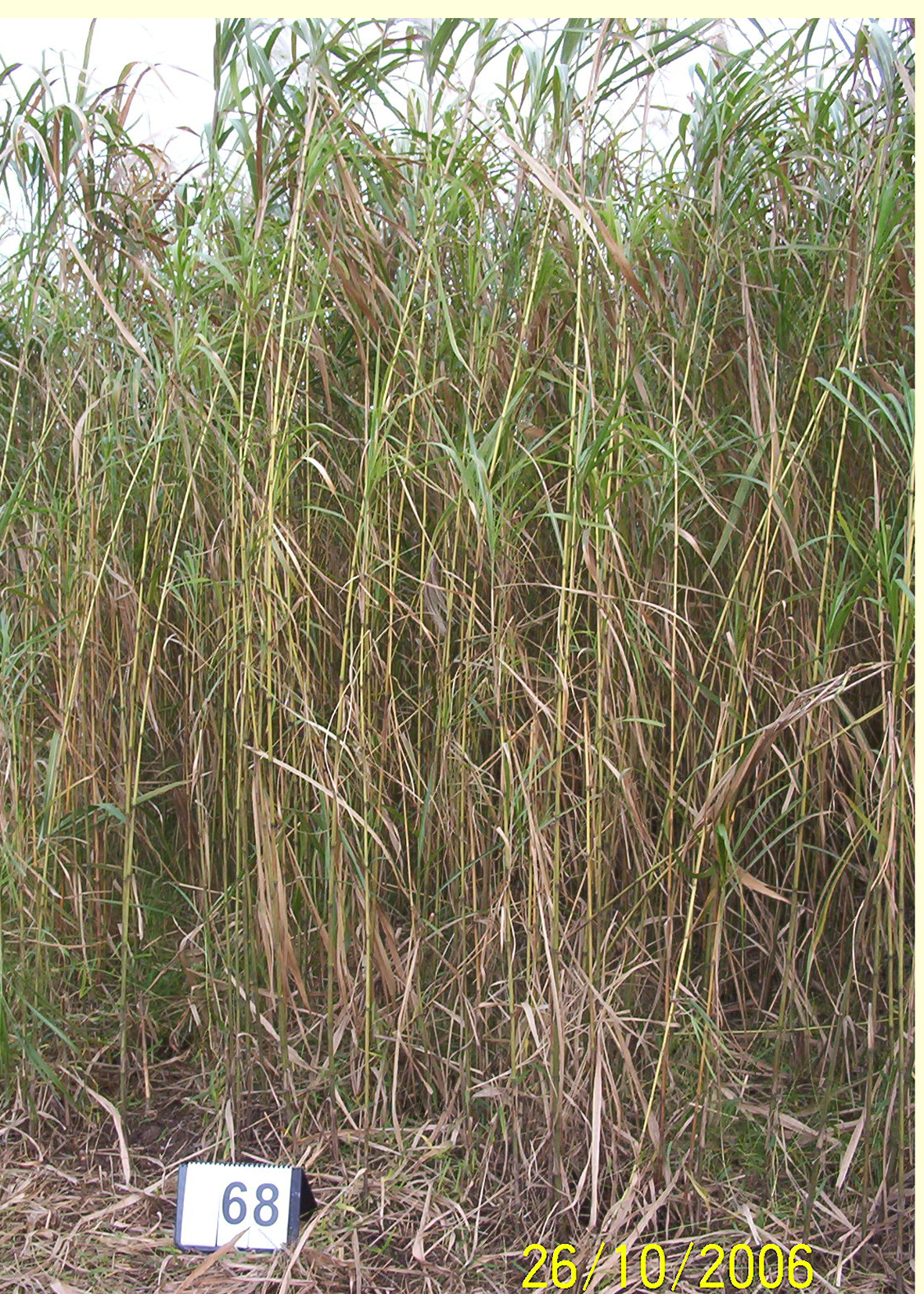

Supplement: mcy231_suppl_Supplementary_Figure_2 [file mcy231_suppl_supplementary_figure_2.jpeg]

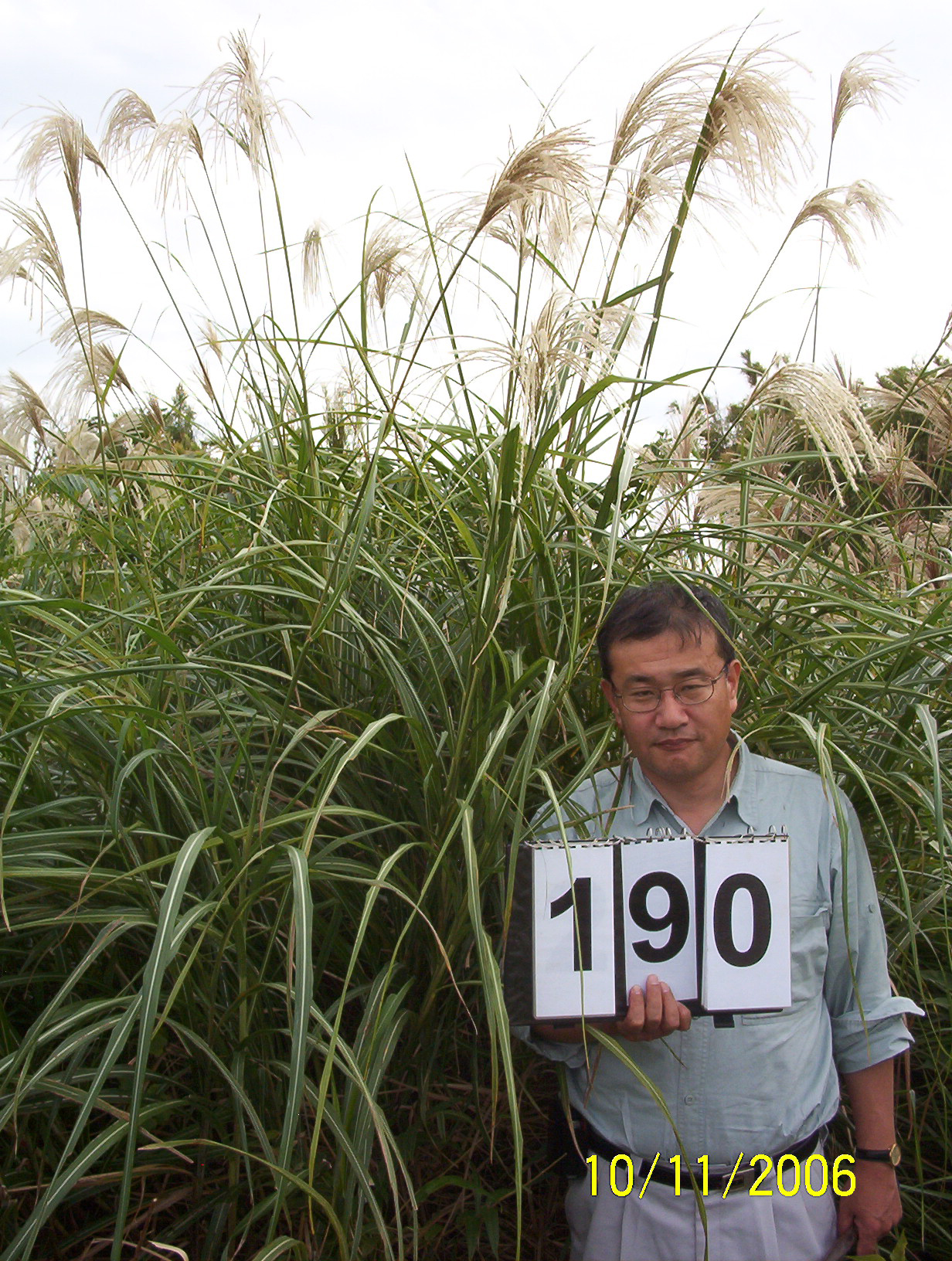

Supplement: mcy231_suppl_Supplementary_Figure_3 [file mcy231_suppl_supplementary_figure_3.jpeg]

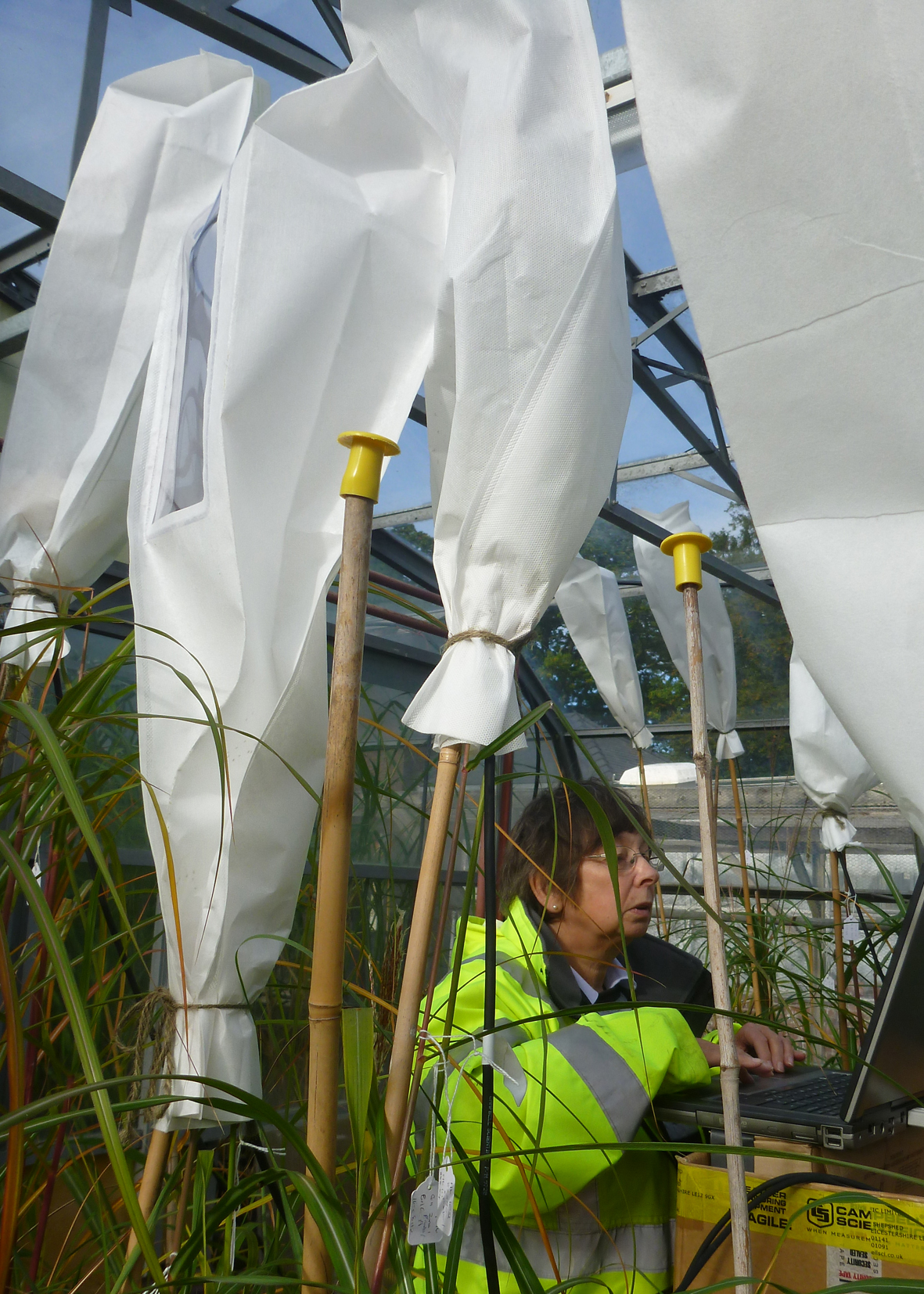

Supplement: mcy231_suppl_Supplementary_Figure_4 [file mcy231_suppl_supplementary_figure_4.jpeg]

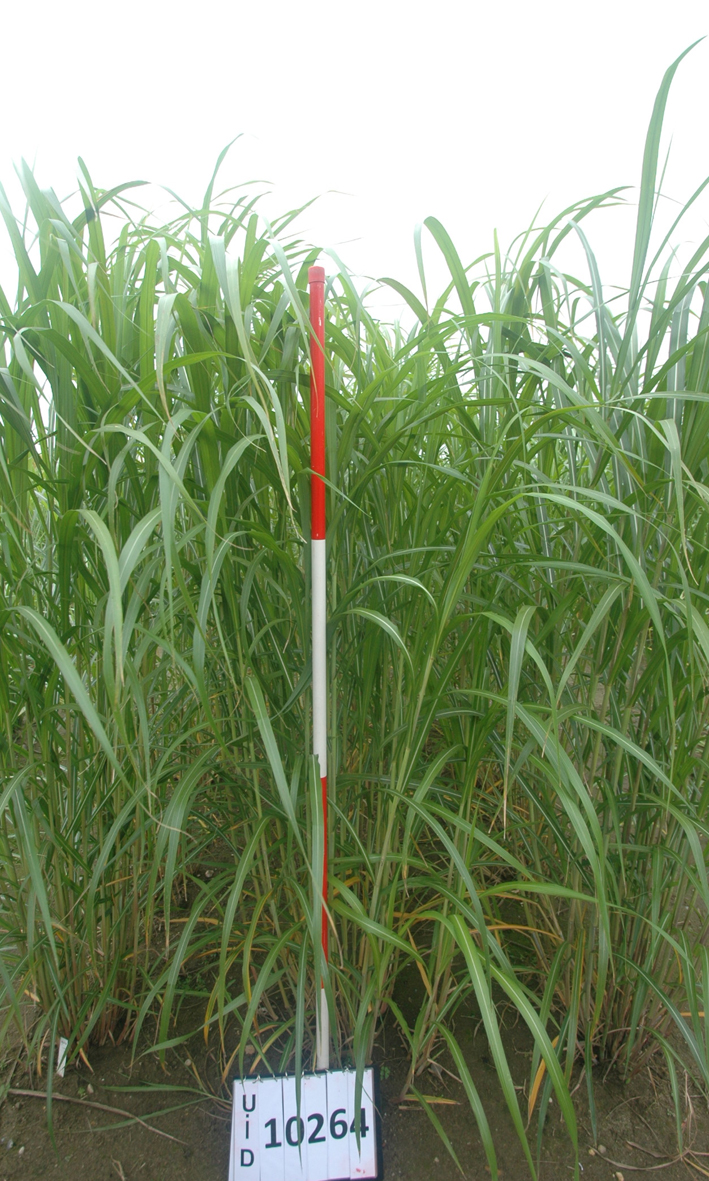

Supplement: mcy231_suppl_Supplementary_Figure_5 [file mcy231_suppl_supplementary_figure_5.jpeg]

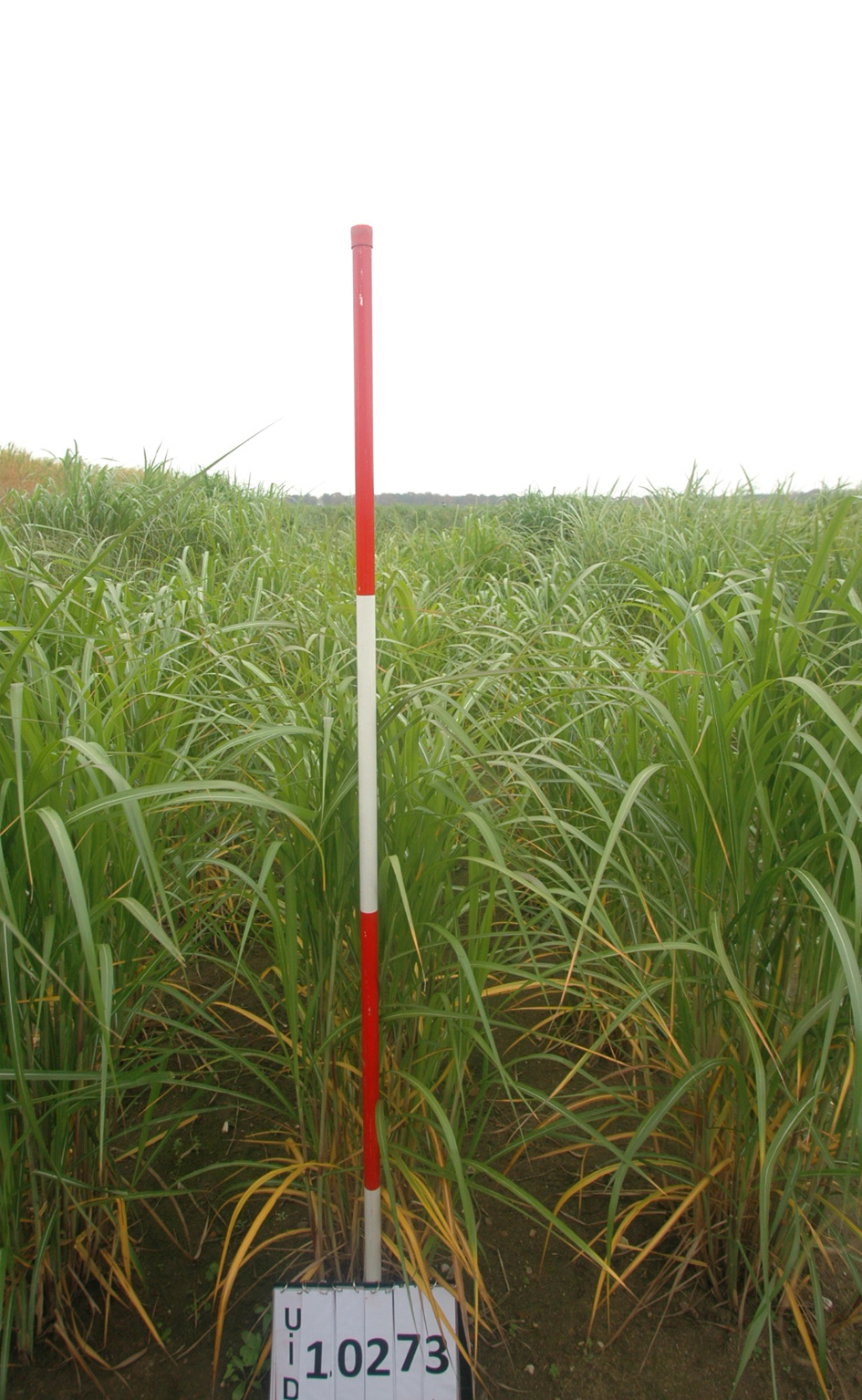

Supplement: mcy231_suppl_Supplementary_Figure_6 [file mcy231_suppl_supplementary_figure_6.jpeg]
