# Supplementary material for: Collecting wild Miscanthus germplasm in Asia for crop improvement and conservation in Europe whilst adhering to the guidelines of the United Nations’ Convention on Biological Diversity
Source: Ann Bot. 2018 Dec 22;124(4):591–604. doi: 10.1093/aob/mcy231 (PMC6821356; doi:10.1093/aob/mcy231)
Supplement: mcy231_suppl_Supplementary_Material_S1 [file mcy231_suppl_supplementary_material_s1.doc]

**S1. Further descriptions of the 1993 Convention for Biological diversity (CBD), 2010 Nagoya Protocol and 2014 Regulation (EU) No 511/2014 on compliance measures for users from the Nagoya Protocol on Access to Genetic Resources and the Fair and Equitable Sharing of Benefits Arising from their Utilization**

***Convention on Biological Diversity (CBD) and Nagoya Protocol***

Since the entry into force of the United Nations Convention on Biological Diversity (CBD) in 1993, any new genetic resources collected need to abide to CBD principles of access and benefit sharing where this is required by national legislation (United Nations, 1993). One of the most significant foundations of CBD is acknowledging that nation states have a sovereign right over their own biological resources and the sustainable use of those resources. CBD Articles 3 and 15 recognise that nation states are responsible for access to and exploitation of their own genetic resources. National governments have the authority to determine access to their genetic resources in order to ensure their ethically- and environmentally- sound use. CBD Article 6 states that national governments should develop national strategies, plans or programmes for the conservation and sustainable use of biological diversity. Article 15 also states that Contracting Parties should provide access to genetic resources for environmentally sound uses. Furthermore, Contracting Parties that decide to determine access to genetic resources should do so through national legislation. As the germplasm collection described here was led by breeders, germplasm was expected to be used for purposes beyond academic research, potentially extending to commercial exploitation. It was therefore crucial that best practices of the CBD, the Bonn Guidelines on Access to Genetic Resources and Fair and Equitable Sharing of the Benefits Arising out of their Utilization (United Nations, 2002) and its supplementary agreement, the Nagoya protocol (see below) for access and benefit-sharing were followed.

The Nagoya Protocol on Access to Genetic Resources and the Fair and Equitable Sharing of Benefits Arising from their Utilization to the Convention on Biological Diversity (Nagoya Protocol) was adopted on 29 October 2010 in Nagoya, Japan and is a supplementary agreement to CBD (Nagoya Protocol, 2010). It provides a transparent legal framework for the effective implementation of CBD’s third objective ‘access and benefit sharing (ABS)’ to support the conservation and sustainable utilisation of biological diversity. It applies to genetic resources and the benefits arising from their utilization where the genetic resources are accessed from Contracting Parties to that Protocol who put in place national legislation. However, where benefits arise from research and development activities involving genetic resources, best practice would suggest that these benefits should be shared with the donor country even in the absence of national legislation or prior to a country becoming a Party to the Nagoya Protocol. The Protocol also covers traditional knowledge associated with genetic resources, with provisions for access, benefit-sharing and compliance. The scheme creates greater legal certainty and transparency for both providers and users of genetic resources by establishing a road map and more predictable conditions for access to and utilisation of genetic resources. Moreover, it helps to ensure benefit-sharing when genetic resources leave the donor. By ensuring benefit-sharing, the Nagoya Protocol incentivises the conservation and sustainable (and ethical) use of genetic resources, and thus enhances the contribution that they make to biodiversity and conservation. The CBD and benefit-sharing have also been recognised by scientists as being able to contribute to a more resilient agriculture under the conditions of climate change (Winge, 2014). They have suggested that through breeding environmentally adaptable and resilient varieties can contribute to agriculture adaptation; and also through benefit-sharing with the Parties contributing the genetic resources, the resulting funding can be used for adaptation and conservation initiatives.

Users of genetic resources, and/or traditional knowledge associated with those genetic resources that fall within the scope of relevant national legislation, are obliged to seek prior informed consent (PIC) and to establish mutually agreed terms (MAT) prior to access and utilisation, i.e. commercial application. Where sharing is subject to MAT, benefits may be in either monetary form, such as royalty payment and licence fees, or non-monetary, such as collaboration in academic research and publication, exchange of genetic resources and expertise, and the sharing of research results.

National legislation is central to the implementation of the Nagoya Protocol. Parties to the Protocol are expected to provide legal clarity for users and providers of genetic resources and associated traditional knowledge. Information about the Parties to the Protocol and relevant legislation can be found at the ABS Clearing-House (ABS-CH, 2015). The ABS-CH, established by Article 14 of the Nagoya Protocol and under Article 18, paragraph 3 of CBD, is a key tool for facilitating the implementation of the Nagoya Protocol, by enhancing legal certainty and transparency in procedures for access and benefit-sharing, and for monitoring the utilization of genetic resources along the value chain, including by means of the internationally recognized certificate of compliance. A list giving the various access and benefit-sharing measures for each individual signatory country can also be found at ABS-CH (2015).

***European Union Regulation (EU) No 511/2014***

The European Union and the UK are party to the CBD and the Nagoya Protocol. Accordingly, Regulation (EU) No 511/2014 of the European Parliament and of the Council on compliance measures for users from the Nagoya Protocol on Access to Genetic Resources and the Fair and Equitable Sharing of Benefits Arising from their Utilization in the Union, was adopted on 16 April 2014 (European Union, 2014). This regulation brings EU law into line with international obligations. It entered into force on 9 June 2014 and all of its provisions applied with effect from 12 October 2015. A list of Designated Competent Authorities under this Regulation can be found in European Union (2014).

In the UK, “Regulatory Delivery” is the enforcement agency for the EU ABS regulations and is expected to engage actively with research communities and commercial sectors that may be affected by the Nagoya Protocol and ABS obligations in order to ensure compliance. As required by the Nagoya Protocol, good practice guidelines and appropriate due diligence approaches for collecting, management and utilisation of genetic resources have been promoted (European Union, 2015). A consultation process has been conducting with UK research and commercial communities and stakeholders regarding the drafting and implementation of guidelines and registration procedures (Defra, 2014).

Access and benefit sharing agreements with donors of genetic resources are keys to commercialisation. However, the implementation of national legislation is still in its infancy even following the agreement (2010) and entry into force (2014) of the Nagoya Protocol. The International Treaty on Plant Genetic Resources for Food and Agriculture (the Plant Treaty; ITPGRFA), a multi-lateral system for ABS, had been put in place for 35 food crops and 29 forage crops (so called Annex 1 crop plants) (Plant Treaty, 2014). However, *Miscanthus* is not included as one of the crop species in the Plant Treaty’s annex 1, although the conservation and use of *Miscanthus* in agriculture is arguably within the Plant Treaty’s scope. Following the agreement of the 2010 Nagoya Protocol, there is an implication that compliance issues relating to the Protocol will increase the need for due diligence and record keeping even for those materials sourced via ITPGRFA and the standard material transfer agreement (SMTA). The SMTA is the model agreement that forms part of the ITPGRFA for the implementation of access and benefit sharing. This development has shown that it has become possible in principle to enter into bi-lateral ABS agreements for ‘non-Annex 1’ crops such as *Miscanthus*. The provisions of guidelines to 2014 EU regulations also allow for users who obtain genetic resources from a collection with registered collection status to become automatically considered to have exercised due diligence.

LITERATURE CITED

**ABS-CH 2015**. The Access and benefit-Sharing Clearing House <https://absch.cbd.int/countries>

**European Union. 2014**. ‘EU Regulation on compliance measures for users from the Nagoya Protocol on Access to Genetic Resources and the Fair and Equitable Sharing of Benefits Arising from their Utilization in the Union’ <http://ec.europa.eu/environment/nature/biodiversity/international/abs/legislation_en.htm>

**Nagoya Protocol. 2010**. ‘The Nagoya Protocol on Access to Genetic Resources and the Fair and Equitable Sharing of Benefits Arising from their Utilization (ABS) to the Convention on Biological Diversity’ <https://www.cbd.int/abs/doc/protocol/nagoya-protocol-en.pdf> ; <https://www.cbd.int/abs/>

**Plant Treaty. 2014**. Crops and Forages of Annex 1: List of crops covered under the Multilateral System, The International Treaty on plant genetic resources for food and agriculture <http://www.planttreaty.org/content/crops-and-forages-annex-1>

**United Nations (UN). 1993**. The Convention on Biological Diversity (CBD) <https://www.cbd.int/convention/>

**United Nations (UN) 2002.** The Bonn Guidelines on Access to Genetic Resources and Fair and Equitable Sharing of the Benefits Arising out of their Utilization. <https://www.cbd.int/doc/publications/cbd-bonn-gdls-en.pdf>

**Winge T. 2014**. Linking access and benefit-sharing for crop genetic resources to climate change adaptation. *Plant Genetic Resources: Characterization*, 1-17. doi:10.1017/S1479262114001038
