# Supplementary material for: Collecting wild Miscanthus germplasm in Asia for crop improvement and conservation in Europe whilst adhering to the guidelines of the United Nations’ Convention on Biological Diversity
Source: Ann Bot. 2018 Dec 22;124(4):591–604. doi: 10.1093/aob/mcy231 (PMC6821356; doi:10.1093/aob/mcy231)
Supplement: mcy231_suppl_Supplementary_Material_S2 [file mcy231_suppl_supplementary_material_s2.doc]

**S2.** Standard operating procedure (SOP) for the import of vegetative rhizomatous material into quarantine in the UK under licence from Defra Plant Health and Seeds Inspectorate (PHSI).

**Standard Operating Procedures for Imported *Miscanthus* spp. and *Triarrhena* spp. to be housed in Compartment 13, Glasshouse C24.**

**Pre-export requirements:** On collection, the *Miscanthus* spp. must be visually inspected for infestations of known pests, fungal infections, phytoplasmas, viroids, viruses and virus-like infections. Only rhizomes from healthy, vigorous plants will be collected. Any rhizomes that show signs of necrotic lesions or patches which may be attributable to invasion by the burrowing nematode (*Radopholus similes*) must be discarded. Rhizomes should be individually packaged and labelled and should be free of soil and all plant debris, wrapped in damp paper and sealed in a polythene bag.

**Transit requirements:** The rhizomes should be ‘double bagged’ and transported in stout, secure and sealed boxes and shipped directly to a quarantine facility at Institute of Biological, Environmental and Rural Sciences (IBERS), UK.

**On arrival:** Packages containing rhizomes under licence must remain sealed before entry into the large compartment (number 13) of quarantine house C24.

After removal from the transit box and the outer polythene bag, but before opening the final polythene bag, visually inspect the package for pests. Any suspect packages should remain sealed and destroyed by incineration or autoclaving.

When the rhizomes are unwrapped inspect the material for fungal pathogens and signs of necrotic lesions or patches (as above); sterilize and discard suspect rhizomes as described below. The rhizomes will be surface sterilized before planting by a brief immersion in 70% alcohol followed immediately by thorough washing in water. Remove roots and any attached particles of soil, and sheath material that is not necessary; shorten the rhizome to the smallest number of nodes required for establishment. Pot in suitable containers using freshly purchased, sterilized compost.

Unwanted plant material, including dead leaf, stem and roots and associated soil should be either removed ‘double bagged’ and autoclaved as described above, or placed in a sealed bag supported by a bucket. The contents should be treated with strong disinfectant (Jeyes fluid) before being disposed of at a designated waste disposal site or incinerated. The same procedures should be applied on completion of any work using plant material remaining under quarantine restrictions. Packaging materials should be bagged and autoclaved.

**Other regulations:** The door to the quarantine compartment is to remain locked and the keys available to named personnel only. A notice stating that quarantined material is being housed within compartment 13 of the glasshouse shall be attached to both the outer and inner door of quarantine house C24, and the door to compartment 13. These notices will state the Defra Plant Health Licence number, the name of staff authorised to enter the facility and to work with the quarantined material, the plant species being grown and a contact name and telephone number.

Once *Miscanthus* and *Triarrhena* are held under licence within compartment 13, it will not be permissible to introduce unlicensed plant material or further plant material held in quarantine under another Defra Plant Health licence.

Access to plant material in quarantine is restricted to the named personnel who shall sign a copy of this SOP to be retained by the IBERS Quarantine Officer. Dated records of all introductions of licenced material must be kept, together with records of any treatment, feeding or crossing of the quarantined materials. Should any plant exhibit signs of disease, this plant should be isolated and kept in a separate, locked, side compartment and inspected regularly. Plants may be returned to the main compartment once it can be deteremined that they are healthy and diseased material should be autoclaved.

When entering, working or leaving the quarantine facility, two doors should not be opened at the same time. Protective laboratory coats and wellington boots, kept within the quarantine house, should be worn at all times when working inside the facility. This protective clothing is never to be removed from the facility.

Plants will be inspected and watered at least once a week.

Integrated pest control should be practised with particular attention to plants which are maintained for long periods of time. If infestations occur, requests for biological or chemical control will be arranged with the glasshouse manager observing strict quarantine procedures. All users must be informed if spraying is planned.

**Plant Health Quarantine**

General Notes on the use of Quarantine House C24.

Permission to bring plant material into the IBERS Quarantine facilities must be obtained through the IBERS Quarantine Officer, who will arrange for a plant health licence from Defra Plant Health, and where applicable in consultation with the IBERS Genetic Manipulation Biological Safety Officer.

The glasshouse has sealed glass and double doors. There is a work area before the machine room which provides filtered air through an underground supply system. Each compartment has filtered outlets. A boost system is provided for hot days and the user can determine the temperature which activates the cooling fans. The compartments are under positive pressure.

Plants pots stand in individual trays or saucers and plants are watered either by capillary matting, by hand or by drip irrigation so that the floor is not wet. Drainage is to a central sump where it is held in the trap for microbe decomposition and overflow to ground supply.

Waste vegetative material will be removed and held in the compartments. If there are any signs of insects or fungi, plants are spray with a cocktail of insecticides and fungicides. All waste material is bagged and Jeyes fluid poured over the material at the strongest recommended solution, in sufficient volume to just wet everything. Trays are washed down and capillary matting, if used, allowed to dry. Waste is transferred to a door lock where it is collected by potting shed staff in an approved manner. Deliveries of potting material etc. are left within the double doors.

Alternatively, waste management facilities (large glasshouse autoclave and immersion bath for pots) are available within the new Transgenic glasshouse and may be used by arrangement with the transgenic glasshouse autoclave supervisor. Materials to be transferred to this facility must be ‘double bagged’ in large autoclave bags, secured at the opening, and suitably labelled before transit from C24 to the Transgenic glasshouse.
