# Supplementary material for: Collecting wild Miscanthus germplasm in Asia for crop improvement and conservation in Europe whilst adhering to the guidelines of the United Nations’ Convention on Biological Diversity
Source: Ann Bot. 2018 Dec 22;124(4):591–604. doi: 10.1093/aob/mcy231 (PMC6821356; doi:10.1093/aob/mcy231)
Supplement: mcy231_suppl_Supplementary_Material_S3 [file mcy231_suppl_supplementary_material_s3.doc]

**Table S3.** Summary of site information including latitudes, longitudes and altitudes, species, ploidy level and countries of collection of 158 collection sites where 303 accessions of germplasm were collected between September-December 2006 in SE Asia.

Details of site information can be found via the UKNPI URL: [https://fusiontables.google.com/DataSource?docid=1e1Eb9ZN8-MKg9f8XZnDnaNaO-d8nQzfbxmqYt3zg#chartnew:id=4](https://fusiontables.google.com/DataSource?docid=1e1Eb9ZN8-MKg9f8XZnDnaNaO-d8nQzfbxmqYt3zg#chartnew:id=4)

**#** *M. Sin*= *M. sinensis*; *M. Sac*= *M. sacchariflorus* *M. Flo*=*M. floridulus* *M. Sac/Sin*=natural hybrid between *M. sacchariflorus* and *M. sinensis.*

** The symbol ‘-‘ indicates that ploidy level is not available. This could be due to the plants have died or to those accessions giving low confidence levels in ploidy tests, or plants that were not tested for ploidy levels because the limited resources at institute.

| **Site** | **Latitude** | **Longitude** | **Altitude (m)** | **Country** | **Species (#)** | **Ploidy (**)** |
| --- | --- | --- | --- | --- | --- | --- |
| S06A1 | 34.31 | 117.95 | 12 | China | *M. Sac* | 3,4 |
| S06A2 | 34.31 | 117.95 | 18 | China | *M. Sac* | 4 |
| S06A3 | 34.29 | 117.95 | 27 | China | *M. Sac* | 2 |
| S06A4 | 28.93 | 120.36 | 280 | China | *M. Sin* | 2 |
| S06A5 | 28.93 | 120.36 | 290 | China | *M. Sin* | 2 |
| S06A6 | 28.20 | 112.91 | 50 | China | *M. Sin* | 2 |
| S06A7 | 28.21 | 112.91 | 45 | China | *M. Sin* | - |
| S06A8 | 28.83 | 112.40 | 33 | China | *M. Sac* | 2,4 |
| S06A9 | 28.81 | 112.41 | 37 | China | *M. Sac* | 2,4 |
| S06A10 | 28.82 | 112.43 | 26 | China | *M. Sac* | 2 |
| S06A11 | 28.83 | 112.43 | 43 | China | *M. Sac* | 2 |
| S06A12 | 28.86 | 112.38 | 28 | China | *M. Sac* | - |
| S06A13 | 28.89 | 112.37 | 21 | China | *M. Sac* | 2 |
| S06A14 | 28.90 | 112.37 | 28 | China | *M. Sac* | 2 |
| S06A15 | 28.90 | 112.37 | 32 | China | *M. Sac* | 2 |
| S06A16 | 28.89 | 112.39 | 26 | China | *M. Sac* | 2 |
| S06A17 | 28.88 | 112.44 | 30 | China | *M. Sac* | 2 |
| S06A18 | 23.15 | 113.35 | 68 | China | *M. Sac* | - |
| S06A19 | 29.43 | 113.06 | 23 | China | *M. Sac* | 2 |
| S06A20 | 29.43 | 113.06 | 28 | China | *M. Sac* | 2 |
| S06A21 | 29.43 | 113.07 | 22 | China | *M. Sac* | 2 |
| S06A22 | 29.43 | 113.07 | 27 | China | *M. Sac* | 2 |
| S06A23 | 37.41 | 118.66 | -1 | China | *M. Sac* | 2 |
| S06A24 | 23.44 | 120.51 | 98 | Taiwan | *M. Flo* | 2 |
| S06A25 | 23.43 | 120.66 | 1242 | Taiwan | *M. Flo* | 2 |
| S06A26 | 23.46 | 120.73 | 1454 | Taiwan | *M. Flo* | 2 |
| S06A27 | 23.50 | 120.80 | 2108 | Taiwan | *M. Flo* | 2 |
| S06A28 | 23.50 | 120.81 | 2291 | Taiwan | *M. Flo* | 2 |
| S06A29 | 23.49 | 120.89 | 2619 | Taiwan | *M. Flo* | 2 |
| S06A30 | 23.89 | 120.90 | 612 | Taiwan | *M. Flo* | 2 |
| S06A31 | 24.01 | 121.12 | 872 | Taiwan | *M. Flo* | 2 |
| S06A32 | 24.10 | 121.19 | 2309 | Taiwan | *M. Flo* | 2 |
| S06A33 | 24.16 | 121.29 | 2990 | Taiwan | *M. Sin* | 2 |
| S06A34 | 24.14 | 121.28 | 3123 | Taiwan | *M. Flo* | 2 |
| S06A35 | 24.02 | 121.13 | 1143 | Taiwan | *M. Flo* | 2 |
| S06A36 | 24.01 | 120.85 | 328 | Taiwan | *M. Flo* | 2 |
| S06A37 | 33.12 | 139.75 | 37 | Japan | *M. Sin* | 2 |
| S06A38 | 33.13 | 139.74 | 116 | Japan | *M. Sin* | 2 |
| S06A39 | 33.07 | 139.80 | 175 | Japan | *M. Sin* | 2 |
| S06A40 | 33.08 | 139.81 | 282 | Japan | *M. Sin* | 2 |
| S06A41 | 33.13 | 139.77 | 504 | Japan | *M. Sin* | 2 |
| S06A42 | 33.13 | 139.80 | 71 | Japan | *M. Sac* | 2 |
| S06A43 | 34.11 | 134.54 | 25 | Japan | *M. Sac* | 3,4 |
| S06A44 | 34.08 | 134.35 | 22 | Japan | *M. Sac* | 4 |
| S06A45 | 33.95 | 133.65 | 342 | Japan | *M. Sin* | 2 |
| S06A46 | 33.94 | 133.64 | 222 | Japan | *M. Sin* | 2 |
| S06A47 | 33.82 | 133.67 | 332 | Japan | *M. Sin* | 2 |
| S06A48 | 33.59 | 133.63 | 12 | Japan | *M. Sin* | 2 |
| S06A49 | 31.89 | 131.40 | 13 | Japan | *M. Sac* | 4 |
| S06A50 | 31.75 | 131.08 | 139 | Japan | *M. Sac* | 4 |
| S06A51 | 32.01 | 130.94 | 273 | Japan | *M. Sac* | 2 |
| S06A52 | 32.04 | 130.85 | 243 | Japan | *M. Sac* | 4 |
| S06A53 | 32.21 | 130.81 | 137 | Japan | *M. Sac/Sin* | 2,4 |
| S06A54 | 32.73 | 130.95 | 484 | Japan | *M. Sac* | 4 |
| S06A55 | 32.71 | 130.98 | 512 | Japan | *M. Sac* | 4 |
| S06A56 | 32.71 | 131.30 | 300 | Japan | *M. Sin* | 2 |
| S06A57 | 32.69 | 131.13 | 582 | Japan | *M. Sac* | - |
| S06A58 | 30.74 | 131.06 | 156 | Japan | *M. Sin* | 2 |
| S06A59 | 30.73 | 131.05 | 70 | Japan | *M. Sin* | 2 |
| S06A60 | 30.72 | 130.98 | 23 | Japan | *M. Sin* | 2 |
| S06A61 | 30.51 | 130.96 | 90 | Japan | *M. Sin* | 2,4 |
| S06A62 | 30.49 | 130.96 | 40 | Japan | *M. Sin* | 2 |
| S06A63 | 30.46 | 130.97 | 175 | Japan | *M. Sin* | 2 |
| S06A64 | 44.20 | 142.05 | 364 | Japan | *M. Sin* | 2 |
| S06A65 | 44.20 | 141.93 | 139 | Japan | *M. Sin* | - |
| S06A66 | 44.27 | 141.70 | 14 | Japan | *M. Sin* | - |
| S06A67 | 44.28 | 141.66 | 7 | Japan | *M. Sac* | - |
| S06A68 | 44.05 | 141.66 | 0 | Japan | *M. Sin* | - |
| S06A69 | 43.90 | 141.71 | 12 | Japan | *M. Sin* | 2 |
| S06A70 | 44.17 | 142.41 | 153 | Japan | *M. Sin* | - |
| S06A71 | 44.19 | 142.34 | 231 | Japan | *M. Sin* | - |
| S06A72 | 43.92 | 142.50 | 211 | Japan | *M. Sin* | - |
| S06A73 | 43.72 | 142.37 | 133 | Japan | *M. Sac* | - |
| S06A74 | 43.60 | 142.47 | 243 | Japan | *M. Sac* | - |
| S06A75 | 43.48 | 142.24 | 115 | Japan | *M. Sin* | 2 |
| S06A76 | 43.56 | 142.04 | 44 | Japan | *M. Sac* | - |
| S06A77 | 43.69 | 142.08 | 114 | Japan | *M. Sin* | - |
| S06A78 | 43.59 | 141.99 | 60 | Japan | *M. Sin* | - |
| S06A79 | 43.62 | 142.00 | 146 | Japan | *M. Sin* | - |
| S06A80 | 43.33 | 141.82 | 16 | Japan | *M. Sin* | - |
| S06A81 | 43.33 | 141.92 | 96 | Japan | *M. Sac* | - |
| S06A82 | 43.33 | 141.93 | 82 | Japan | *M. Sin* | - |
| S06A83 | 43.38 | 141.89 | 80 | Japan | *M. Sin* | 2 |
| S06A84 | 43.16 | 141.76 | 59 | Japan | *M. Sac* | - |
| S06A85 | 43.17 | 141.82 | 53 | Japan | *M. Sin* | 2 |
| S06A86 | 43.15 | 141.75 | 26 | Japan | *M. Sac* | - |
| S06A87 | 43.55 | 143.45 |  | Japan | *M. Sin* | - |
| S06A88 | 43.26 | 141.59 | 15 | Japan | *M. Sac* | - |
| S06A89 | 43.17 | 141.43 | 11 | Japan | *M. Sac* | 4 |
| S06A90 | 42.95 | 141.79 | 60 | Japan | *M. Sac* | - |
| S06A91 | 43.25 | 141.71 | 17 | Japan | *M. Sac* | - |
| S06A92 | 43.24 | 141.35 |  | Japan | *M. Sin* | 2 |
| S06A93 | 43.16 | 141.27 | 1 | Japan | *M. Sac* | 4 |
| S06A94 | 43.01 | 141.41 | 56 | Japan | *M. Sin* | - |
| S06A95 | 43.04 | 141.29 | 146 | Japan | *M. Sin* | 2 |
| S06A96 | 43.00 | 141.37 | 88 | Japan | *M. Sin* | 2 |
| S06A97 | 42.99 | 141.45 | 79 | Japan | *M. Sac* | - |
| S06A98 | 42.90 | 141.57 | 40 | Japan | *M. Sac* | - |
| S06A99 | 42.91 | 141.73 | 17 | Japan | *M. Sac* | - |
| S06A100 | 43.21 | 140.94 | 44 | Japan | *M. Sin* | - |
| S06A101 | 43.19 | 140.96 | -20 | Japan | *M. Sin* | - |
| S06A102 | 43.30 | 140.55 | 128 | Japan | *M. Sin* | - |
| S06A103 | 43.33 | 140.36 | 66 | Japan | *M. Sin* | - |
| S06A104 | 43.06 | 140.50 | 30 | Japan | *M. Sin* | - |
| S06A105 | 42.95 | 140.56 | 171 | Japan | *M. Sin* | - |
| S06A106 | 42.84 | 140.65 | 307 | Japan | *M. Sin* | - |
| S06A107 | 42.87 | 140.60 | 569 | Japan | *M. Sin* | 2 |
| S06A108 | 42.18 | 140.44 | 15 | Japan | *M. Sin* | 2 |
| S06A109 | 42.30 | 140.27 | -1 | Japan | *M. Sac* | - |
| S06A110 | 42.40 | 140.30 | 0 | Japan | *M. Sin* | - |
| S06A111 | 42.58 | 140.45 | 2 | Japan | *M. Sin* | - |
| S06A112 | 42.47 | 140.20 | 121 | Japan | *M. Sin* | - |
| S06A113 | 42.60 | 140.65 | -3 | Japan | *M. Sin* | - |
| S06A114 | 42.56 | 140.80 | 187 | Japan | *M. Sin* | - |
| S06A115 | 42.62 | 140.79 | 321 | Japan | *M. Sin* | 2 |
| S06A116 | 42.78 | 140.80 | 264 | Japan | *M. Sin* | - |
| S06A117 | 41.72 | 141.01 | 25 | Japan | *M. Sin* | 2 |
| S06A118 | 41.74 | 140.92 | 15 | Japan | *M. Sin* | - |
| S06A119 | 41.91 | 140.97 | 37 | Japan | *M. Sin* | - |
| S06A120 | 41.78 | 140.61 | 11 | Japan | *M. Sin* | - |
| S06A121 | 41.60 | 140.35 | 28 | Japan | *M. Sin* | - |
| S06A122 | 41.40 | 140.19 | 20 | Japan | *M. Sac/Sin* | 2 |
| S06A123 | 41.40 | 140.22 | 25 | Japan | *M. Sin* | 2 |
| S06A124 | 43.92 | 142.50 | 211 | Japan | *M. Sac/Sin* | - |
| S06A125 | 41.79 | 140.08 | 11 | Japan | *M. Sac/Sin* | - |
| S06A126 | 41.90 | 140.14 | 34 | Japan | *M. Sin* | - |
| S06A127 | 41.93 | 140.30 | 20 | Japan | *M. Sin* | - |
| S06A128 | 41.98 | 140.67 | 127 | Japan | *M. Sin* | - |
| S06A129 | 42.07 | 140.80 | 24 | Japan | *M. Sin* | - |
| S06A130 | 43.45 | 144.58 | 157 | Japan | *M. Sac* | - |
| S06A131 | 43.53 | 144.49 | 253 | Japan | *M. Sin* | - |
| S06A132 | 43.62 | 144.45 | 172 | Japan | *M. Sin* | - |
| S06A133 | 43.66 | 144.25 | 429 | Japan | *M. Sin* | - |
| S06A134 | 43.75 | 143.72 | 235 | Japan | *M. Sin* | - |
| S06A135 | 43.89 | 144.00 | 86 | Japan | *M. Sac* | - |
| S06A136 | 43.88 | 144.06 | 128 | Japan | *M. Sin* | - |
| S06A137 | 43.55 | 143.45 |  | Japan | *M. Sac* | - |
| S06A138 | 39.70 | 141.14 |  | Japan | *M. Sin* | - |
| S06A139 | 36.20 | 140.28 |  | Japan | *M. Sin* | - |
| S06A140 | 37.02 | 140.12 |  | Japan | *M. Sin* | 2 |
| S06A141 | 36.11 | 137.96 |  | Japan | *M. Sin* | 2 |
| S06A142 | 36.64 | 138.19 | 1129 | Japan | *M. Sin* | 2 |
| S06A143 | 35.89 | 138.42 | 1078 | Japan | *M. Sin* | - |
| S06A144 | 35.91 | 138.41 | 1118 | Japan | *M. Sin* | - |
| S06A145 | 35.92 | 138.44 | 1205 | Japan | *M. Sin* | - |
| S06A146 | 35.76 | 138.45 |  | Japan | *M. Sin* | 2 |
| S06A147 | 35.82 | 138.37 |  | Japan | *M. Sin* | - |
| S06A148 | 28.89 | 112.10 | 26 | China | *M. Sac* | 2 |
| S06A149 | 28.91 | 112.19 | 28 | China | *M. Sac* | 2 |
| S06A150 | 28.92 | 112.15 | 28 | China | *M. Sac* | 2 |
| S06A151 | 28.92 | 112.16 | 32 | China | *M. Sac* | 2 |
| S06A152 | 28.91 | 112.12 | 33 | China | *M. Sac* | 2 |
| S06A153 | 28.90 | 112.12 | 37 | China | *M. Sac* | 2 |
| S06A154 | 28.90 | 112.13 | 24 | China | *M. Sac* | 2 |
| S06A155 | 29.07 | 111.69 | 42 | China | *M. Sac* | 2 |
| S06A156 | 23.20 | 120.00 |  | Taiwan | *M. Flo* | - |
| S06A157 | 23.87 | 120.00 |  | Taiwan | *M. Flo* | - |
| S06A158 | 23.93 | 120.00 |  | Taiwan | *M. Flo* | - |
